# Tracking group identity through natural language within groups

**DOI:** 10.1093/pnasnexus/pgac022

**Published:** 2022-06-24

**Authors:** Ashwini Ashokkumar, James W Pennebaker

**Affiliations:** Polarization and Social Change Lab, 450 Jane Stanford Way Building 120, Room 201, Stanford, CA 94305, USA; Department of Psychology, University of Texas Austin, 108 E. Dean Keeton, Austin, TX 78712-0187, USA

**Keywords:** group identity, group processes, naturalistic observation, language analysis, LIWC

## Abstract

To what degree can we determine people's connections with groups through the language they use? In recent years, large archives of behavioral data from social media communities have become available to social scientists, opening the possibility of tracking naturally occurring group identity processes. A feature of most digital groups is that they rely exclusively on the written word. Across 3 studies, we developed and validated a language-based metric of group identity strength and demonstrated its potential in tracking identity processes in online communities. In Studies 1a–1c, 873 people wrote about their connections to various groups (country, college, or religion). A total of 2 language markers of group identity strength were found: high affiliation (more words like *we, togetherness*) and low cognitive processing or questioning (fewer words like *think, unsure*). Using these markers, a language-based unquestioning affiliation index was developed and applied to in-class stream-of-consciousness essays of 2,161 college students (Study 2). Greater levels of unquestioning affiliation expressed in language predicted not only self-reported university identity but also students’ likelihood of remaining enrolled in college a year later. In Study 3, the index was applied to naturalistic Reddit conversations of 270,784 people in 2 online communities of supporters of the 2016 presidential candidates—Hillary Clinton and Donald Trump. The index predicted how long people would remain in the group (3a) and revealed temporal shifts mirroring members’ joining and leaving of groups (3b). Together, the studies highlight the promise of a language-based approach for tracking and studying group identity processes in online groups.

Significance Statement Millions of people participate in identity-based social media communities (e.g., Reddit's political communities). The rich language and behavioral data available from these groups makes them a treasure trove for studying naturally occurring group dynamics. But how do we capture people's group identities from unstructured social media data? The current report presents evidence that people's group identities leave traces in the language they use. Specifically, across diverse groups, the language of people with strong identities was marked by (a) a higher focus on affiliation, and (b) lower uncertainty or questioning. Using the two identified language markers, the current work formulated a language-based metric of group identity strength that can track identity processes in large online communities.

## Introduction

What are the fundamental social and psychological processes that are associated with the ways people connect with their school, church, nation, or political candidate? At a broader level, how can we tell when people identify with or feel committed to these groups? What signals do people give off about their unwavering allegiance to groups that others may use to guide their own behaviors? The current project attempts to identify the underlying linguistic fingerprints of people's group identities. By finding such fingerprints, we should be able to identify the people who are most likely to actively contribute to, as opposed to disengage with or leave a group, and at the same time, gain a better understanding of the process through which people's group identities are developed.

Decades of research have revealed that the strength of people's group identities influences their cognitions and behaviors, producing outcomes ranging from motivated reasoning (1) to collective action (2) and violence (3). The rise of social media has led to an explosion of identity-based online communities, wherein people with shared identities (e.g. partisan identity) come together and engage with each other. These communities make for a rich source of language and behavioral data for social scientists to study identity processes “in the wild.” However, the large amounts of real-world behavioral data available from online communities remain largely untapped by identity researchers because little is known about how identity is expressed in people's natural language. The current research introduces a language-based approach for tracking and understanding group identity strength in naturalistic group settings, thereby opening new possibilities for the study of contemporary group processes.

A potential approach to capture group identity strength in the online context is through the analysis of people's language. People's language contains traces of their psychological states (4–6), which can be used to track social and group dynamics. Of particular interest are studies showing that people's individual identities get expressed in their language (7). Some studies have tracked the language markers of phenomena somewhat related to people's social identities, for instance, their moral values based on the Moral Foundations Theory (8) and political ideology (9). Researchers interested in group processes have examined how group members align their linguistic style (10) and content (11) with that of fellow members, but no studies have directly examined the language markers of group identity. Identifying the natural language correlates of group identity strength would not only provide a way to measure group identity from language, but also illuminate naturally occurring psychological processes in group settings. The current research sought to (a) develop and validate a linguistic metric of group identity strength that can be applied to unstructured text data, and (b) demonstrate the metric's potential for capturing group identity in large social media groups.

The group identity literature has a rich history. Since Tajfel introduced social identity theory in the 1970s,  several approaches have illuminated the different features of group identity including activation of the social self (12), intragroup ties (3, 13), uncertainty reduction (14), identity performance and engagement (15), group-level emotions (16), and so on. Because most of these features have been measured by relying on people's self-reports, we were particularly interested in finding which ones might be manifested in everyday language. As described below, we identified 4 facets—2 social, 1 cognitive, and 1 behavioral—which have strong theoretical and empirical links to group identity. See SOM-III for a detailed discussion of why these facets were selected but not others (e.g. emotional facets). The set of identified candidate language markers of group identity strength was then narrowed down based on correlations with validated group identity scales. The 4 candidate language markers were measured from language using prevalidated dictionaries from the text analysis program Linguistic Inquiry and Word Count (LIWC; (17). Of the 4 theory-based candidate language markers identified, the ones that were robustly associated with self-reported group identity strength were then used to develop a language-based metric of group identity strength.

High affiliation. Having a strong group identity, almost by definition, is associated with greater feelings of connectedness with other group members. The classic social identity theory posits that people feel a sense of shared self with others who are similar to them and who belong to their group (18). More recently, identity fusion theory emphasized the strong intragroup bonds that arise from people's group identities (3). This greater feeling of connectedness or we-ness may manifest in use of language focusing on such connections (19), for instance, first person plural pronouns (e.g. we and us) that refer to one's collective self and other affiliation-related words (e.g. together, love).

Low self-focus. A greater focus on one's we-ness may go hand in hand with a drop in their sense of I-ness. Self-categorization theory posits that the salience of one's group identity is associated with “depersonalization,” characterized by an eclipsing of the personal self by the social identity (12). People with a strong identity may, therefore, display decreased levels of self-focus in contexts where their group identity is salient, which can be measured using first-person singular pronouns (e.g. I and me).

High certainty (or low questioning). Several lines of work converge on the notion that group identity is associated with high certainty and low uncertainty. Hogg's classic uncertainty–identity theory argues that identifying with a group reduces epistemic uncertainty. (14). Aligned with this theory, studies show that strong political identities are characterized by feelings of high certainty and low doubt (20, 21). Other studies find that people with strong religious identities rely on their first intuitions, as opposed to engaging in deeper reflection or questioning (22), arguably a marker of certainty. Studies on language show that when people feel uncertain or when they are dealing with unresolved issues, they use more “cognitive processing” words associated with causation (e.g. because, reason), self-reflection (e.g. understand, think), uncertainty (maybe, perhaps), and so on to actively question and work through the issues (23–25). Low use of such cognitive processing words, indicating that the author is not having to engage in active cognitive processing to work through any issues, could indicate lower uncertainty. Combining these literatures, it was expected that people with stronger identities would feel greater certainty on group-related issues, decreasing their need for using cognitive processing words to work through issues. Strong identity should then be negatively associated with use of cognitive processing words.

High engagement. People with strong identities may naturally be most motivated to engage with group-related issues, which has been observed with a range of group-related behaviors (26). Given the current focus on group members’ language, a natural behavioral indicator of group-related engagement is the degree to which group members talk in group-related contexts. For instance, group identity may manifest in a greater tendency to engage in conversations with fellow group members. When given a chance to talk about a group, people with strong group identities may display greater engagement by simply saying more. The number of words spoken could be used as a proxy for engagement. We expected that people with stronger group identities would use more words when talking about the group or in conversations with fellow-members.

The current research sought to evaluate the above-described facets of group identity strength in language across a range of groups. While different types of groups can produce different effects on people's language, in line with the social identity literature examining many types of groups—sports, political, ideological, and even minimal groups, the current work assumes that some core identity processes operate across diverse groups. The goal was to track the language markers of the psychological state of identifying with a group without regard for group-specific topics. In Study 1, we analyzed 3 samples of essays that survey participants wrote about their connections with 3 social groups—country, religion, or college, and evaluated the 4 hypothesized language-based dimensions against established self-report measures of group identity strength. A total of 2 consistent language correlates of group identity emerged from the analysis: high affiliation and low questioning. Using these 2 dimensions, a composite language index—which we call *unquestioning affiliation—*was developed. In Study 2, using an archival sample of college student essays, we validated the developed index against both self-reported university identity strength and an important behavioral outcome: future retention in college.

In Study 3, unquestioning affiliation was measured from conversations occurring within 2 large political groups on Reddit: supporters of the Presidential candidates in the 2016 US presidential election, Donald Trump and Hillary Clinton. We first examined whether the index predicted how long a member would go on to stay in the groups (Study 3a). Long-term, committed group members were expected to use language indicating higher levels of unquestioning affiliation. Second, we tracked temporal changes associated with members’ joining and leaving of online groups (Study 3b). Specifically, we tested if the levels of unquestioning affiliation that members expressed in the Trump and Clinton communities increased after they joined the groups and decreased prior to leaving.

## Studies 1a, 1b, and 1c: Developing a Language-Based Metric of Group Identity Strength

### Methods

To identify the language markers of group identity strength, 3 samples of US residents were recruited from Amazon's Mechanical Turk (MTurk). The final samples included 247 participants in Study 1a, 372 participants in Study 1b, and 250 participants in Study 1c (see SOM-V and Table S1 [Supplementary Material] for demographics, exclusions, and other methodological details).

Participants wrote for 6–8 minutes about their connection to the United States (1a; e.g. “. . .write about your relationship with America. . .”), their religion (1b), or their college (1c). Participants then rated several items on 7-point scales (1* = Strongly Disagree*; 7 = *Strongly Agree*) including the verbal identity fusion scale (e.g. “I am one with America”; (27) measuring group identity strength and also items measuring their intentions to engage in progroup behaviors (e.g. “If I found out that a student from < college name > met with an accident, I would be willing to donate blood to help the student.”).

For only Study 1c, we obtained judge ratings from an undergraduate research assistant who was blind to the hypothesis. The student read about identity fusion theory and then rated 100 essays on the strength of the author's college or university identity. Excluding 3 essays for which no ratings were provided, the ratings were positively correlated with self-reported fusion (*r*(95) = 0.60 and *P* < 0.001).

Participants’ essays were analyzed using LIWC2015 (17). LIWC is a text analysis program that captures various themes and psychological states from text using prevalidated dictionaries. A total of 4 dimensions were analyzed using the corresponding LIWC dictionaries (i) affiliation, (ii) self-focus or I-words, (iii) questioning or cognitive processing, and (iv) engagement or word count (see SOM-IV for LIWC details).

### Results

Correlations between self-reported measures and language indices are presented in Table 1. Across the 3 samples, self-reported identity fusion and progroup behavior were correlated positively with affiliation and negatively with cognitive processing. The correlations with word count and self-focus were neither consistent across the samples nor with our hypotheses (also see Figure S1 [Supplementary Material] in the SOM).

**Table 1.** Correlations of self-reported and language indices in Studies 1a, 1b, and 1c.

Self-reported measures	Affiliation	Self-focus (I-words)	Questioning (cognitive processing)	Engagement (word count)
Study 1a: USA (*N* = 247)				
Identity fusion with group	0.19**	−0.001	−0.14*	−0.16*
Progroup behavior	0.21***	−0.03	−0.10	−0.12*
Study 1b: religion (*N* = 372)				
Identity fusion with group	0.14**	0.06	−0.31***	0.10*
Progroup behavior	0.16**	0.13**	−0.24***	0.003
Study 1c: college (*N* = 250)				
Identity fusion with group	0.25***	0.09	−0.17**	−0.19**
Progroup behavior	0.33***	0.13*	−0.15*	−0.17**

*Note:* *indicates *P* < 0.05. **indicates *P* < 0.01. ***indicates *P* < 0.001.

Because affiliation was positively associated and cognitive processing was negatively associated with the self-reported identity measures across the 3 samples, we computed a composite measure of the 2 dimensions. We reverse-scored the cognitive processing score, and added up the standardized dimensions (i.e. = z(affiliation)—z(cognitive processing)) to compute a language-based index capturing the psychological state of *unquestioning affiliation*. Note that affiliation and cognitive processing were either not correlated with each other or negatively correlated (average *r* = −0.16, ranging from −0.33 to +0.02). The Guttman split half reliabilities of the unquestioning affiliation index across the 3 samples were 0.47, 0.56, and 0.43, respectively.

The unquestioning affiliation index was positively associated with self-reported identity fusion and progroup behavior (0.21 < *r*s < 0.31). This effect remained robust controlling for demographics (gender, race, age, education level, and political orientation) (1a: *β* = 0.21, *P* = 0.002; 1b: *β* = 0.23, *P* < 0.001; and 1c: *β* = 0.25, *P* < 0.001). Providing evidence for divergent validity, none of the demographic variables were consistent significant predictors across the studies (see Table S3 [Supplementary Material] in the SOM for the relevant statistics). Finally, the judge ratings obtained in Study 1c were positively associated with our unquestioning affiliation index (*r*(95) = 0.32, *P* = 0.002.

In sum, as illustrated by the examples in Table 2 and Table S2 (Supplementary Material) in SOM-V, across essays about diverse types of groups, group identity strength was consistently associated with language indicating high affiliation and low questioning, what we term unquestioning affiliation. Given this evidence is based on self-reported identity strength, it is important to assess whether linguistic expressions of unquestioning affiliation can predict real-world behavioral outcomes.

**Table 2.** Excerpts from sample responses with low and high unquestioning affiliation scores. Words from the affiliation dictionary are in green font, and words in the cognitive processing dictionary are in red font.

High unquestioning affiliation	Low unquestioning affiliation
“I **feel** a bond with **members** of my **church family**. I **feel** unending support and **love** from them. I enjoy being there for my **church family** and volunteering as well. It gives me a **sense** of **fellowship** and giving to **others**. It **helps** me **feel** closer to my **church** and to god. . . I feel **we** are kind individuals and as a whole **we try** to do **our** best. . .” (Study 1b)	“I'm just **not sure if** there is a God **or not**. I **can't** help myself. I **guess** I don't **admit** it enough **but sometimes** the **curiosity** does consume me to the point I lose sleep. I **want** to **believe**. I **believe** I **believe**. I'm just **not sure if** I **believe**. If that **makes any sense** . . . I am so **unsure** and so **confused** myself, I just don't **know** what to do **most** of the time.” (Study 1b)

Interestingly, our hypotheses regarding word count and I-words were not confirmed. In fact, word count was negatively correlated with self-reports of group identity in 2 samples. A possible explanation is that some weakly identified participants were motivated to vent out their strong negative feelings about their group. This seemed to be qualitatively true in Study 1c, wherein several participants were disappointed with their college experience and with Study 1a participants who were unhappy with the outcome of the 2016 election when the study was conducted. It is possible that word count captures engagement only in conversational contexts. We are unsure why there was no effect of I-words. This may have to do with the wording of the writing prompt or it could indicate that group members’ personal identities remain activated when their social identity is activated (3).

## Study 2: Testing Unquestioning Affiliation With a Behavioral Outcome: College Retention

Study 2 sought to replicate Study 1’s main finding and additionally assess if the unquestioning affiliation index could predict an important real-world *behavior*. Following evidence that self-reported identity strength with university predicts future college retention (28), we tested whether unquestioning affiliation measured from students’ stream of consciousness essays could predict college retention.

### Methods

Study 2 relied on archival data collected from students (*N* = 1,520) enrolled in an Introduction to Psychology online course taught at a large university. After exclusions, the sample contained 1,512 participants (*M_age_* = 18.81; *SD_age_* = 1.69; 61.8% female, 38.94% White; and 64.0% freshmen) from 2 cohorts: Fall 2015 (*N* = 1,082) and Spring 2016 (*N* = 430). Students completed writing assignments and surveys as part of the course curriculum, which were used for research after obtaining students’ consent. The research methods were approved by the Institutional Review Board of the authors’ institution (see SOM-II for more details on ethics approval).

Participants completed a stream-of-consciousness writing exercise (see (29) for details; average word count = 709). Even though students were not prompted to write about their college, most students did so. The most used words were “time,” “people,” “friend,” “class,” and “college.” A total of 2 months later, participants also completed the identity fusion scale (27) indicating their fusion with university. Because the fusion items and response scales used varied across the semesters, the fusion scores were standardized within each semester (see SOM-VII for descriptive statistics). Students’ enrollment status in the university recorded approximately a year after the above-listed measures were collected was used as an indicator of whether the student stayed in the university. Overall, 7.94% of the sample left the university. Note that a subset of this dataset was previously used in an article by Talaifar et al. (28) exploring the links between self-reported fusion and college retention.

### Results

As in Study 1, unquestioning affiliation scores were computed for each student (i.e. = z[affiliation]—z[cognitive processing]). More detailed analyses of the separate components can be found in SOM-VIII. Replicating the finding from Study 1, unquestioning affiliation expressed in language was positively associated with self-reported fusion with university, *r*(1510) = 0.11; *P* < 0.001. This effect remained robust (*β* = 0.11 and *P* < 0.001) when controlling for participant's cohort, SAT scores, and demographic variables including gender, age, and ethnicity.

A logistic binomial regression was then employed to test whether unquestioning affiliation predicted retention 1 year later, which it did (OR = 1.19, 95% CI = [1.05, 1.36], Wald *χ ^2^* = 7.10, and *P* = 0.008). To illustrate, of the students whose essays received unquestioning affiliation scores over 1 SD above the mean, 95.09% stayed in university after a year as opposed to 89.64% of the students who scored a standard deviation below the mean. This effect was robust when tested with controls (OR = 1.18 and *P* = 0.02). Sample text samples are provided in SOM-VII.

In sum, language expressions of unquestioning affiliation were associated with self-reported group identity and, more notably, with an important group-related behavioral outcome that is difficult to predict: retention in college. These patterns are particularly noteworthy given that students were asked to simply write about their thoughts and feelings while in the university setting with no explicit demand to write about their university identity. The evidence so far relied on analysis of essays that participants wrote as part of surveys. The true test of the validity and utility of our inference that unquestioning affiliation captured in language is a marker of group identity strength is in naturally occurring interactions within real-world identity-based groups. In other words, when people are engaging in conversations with fellow group members, their identities should manifest in the form of linguistic expressions of unquestioning affiliation, which was tested in Study 3.

## Study 3: Applying the Unquestioning Affiliation Index to Large Social Media Political Groups

Unlike in Studies 1 and 2 wherein we analyzed essays that participants wrote about their groups, Study 3 applied the unquestioning affiliation index to naturalistic conversations in 2 large political identity-based groups on Reddit. We sought to determine if unquestioning affiliation measured from conversational language would predict staying in vs. dropping out of the groups. Reddit is a popular social media website on which users participate in discussion-based communities. Comments posted on 2 Reddit groups corresponding to the supporters of the Presidential candidates in the 2016 US presidential election, Donald Trump (The_*Donald*) and Hillary Clinton (*hillaryclinton*), were examined. All the reported findings replicated in a parallel analysis conducted with Bernie Sanders supporters (see Figures S10 and S11 [Supplementary Material] in SOM-XIII). Partisan groups were selected because partisan identities are often expressed and signaled on social media, especially around elections when competition is most intense.

The analysis of conversations occurring within *The_Donald* and *hillaryclinton* examined whether the language-based unquestioning affiliation index reflects (a) individual differences and (b) within-person temporal changes in group identity strength. First, does unquestioning affiliation measured from naturalistic conversations predict retention in the 2 online groups? In line with findings from Study 2, it was expected that strongly committed, long-term members would use language reflecting higher levels of unquestioning affiliation. Second, within-person changes in unquestioning affiliation were tracked over time. It was expected that after joining *The_Donald* or *hillaryclinton*, the longer members stayed in the group and got socialized, the more unquestioning affiliation they would express (11) and in parallel, that unquestioning affiliation would drop as they approached their departure from the group (28). Separate analyses for affiliation and questioning are reported in the SOM (see Figures S5, S6, S8, and S9, Supplementary Material).

## Study 3a: Does the Unquestioning Affiliation Index Predict Tenure in Online Groups?

On entering a political subreddit, it was predicted that new members whose posts reflected greater unquestioning affiliation would remain active in the group longer than others.

### Methods

Comments posted on *The_Donald* since the inception of the group (July 2015) until February 2018 were included. In the case of *hillaryclinton*, comments posted since the group's inception (March 2013) were included only up until September 2017, because the group became inactive by mid-2017 after Clinton lost the 2016 elections. Each user's membership duration was computed as the total number of days on which they posted at least 1 comment on the group. Over 50% of the groups’ members posted for only a couple of days before leaving. The samples were grouped into 3 categories based on membership duration: Members who contributed for 1–5 days (*N*_The_Donald_ = 147,163; *N_hillaryclinton_* = 19,287), 6–40 days (*N*_The_Donald_ = 62,269; *N_hillaryclinton_* = 5,140), and 41 or more days (*N*_The_Donald_ = 35,249; *N_hillaryclinton_* = 1,676). In SOM-XI, we provide the rationale for this grouping (also see Table S5 and Figure S2, Supplementary Material). The findings remained robust treating membership duration as a continuous variable. The comments were aggregated into text files such that each text file contained the comments posted by a single person on a particular day. The analyses excluded texts that had fewer than 25 words, were not in English, or were posted by bots. As discussed in SOM-X, our findings remained robust to alternate exclusion criteria. The final samples contained 2,965,841 text files from 244,681 members on *The_Donald* and 184,202 text files from 26,103 members on *hillaryclinton*. LIWC scores were obtained for the texts and standardized across users within each date to account for variations in the joining date of members. Each user's average unquestioning affiliation score was computed. SOM-XV explores topic-based differences in the language of long-term vs. short-term members.

### Results

**Fig. 1.**
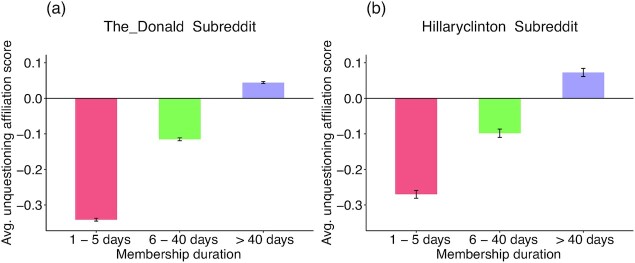
(a) and (b). Average unquestioning affiliation scores among short-, medium-, and long-term members of *The_Donald* (1a) and *hillaryclinton* (1b). Group members who stayed in the group longer conveyed higher levels of unquestioning affiliation in their language on average. Error bars represent confidence intervals.

A one-way anova tested the effect of membership duration on members’ average unquestioning affiliation scores. Note that all effects reported in Study 3 are significant at *P* **<** 0.001 unless noted otherwise. As shown in Fig. 1(a) and (b), members who stayed in the group longer expressed higher levels of unquestioning affiliation on average (*The_Donald*: *d*_>40 vs. 1–5 days_ = 0.28; *hillaryclinton*: *d*_>40 vs. 1–5 days_ = 0.23). When membership duration was treated as a continuous variable, it was still positively associated with unquestioning affiliation (*The_Donald*: Spearman's *ρ* = 0.15; *hillaryclinton*: *ρ* = 0.12; see Figure S3 [Supplementary Material] in the SOM). SOM-XIII shows parallel effects in *SandersForPresident*). Interestingly, the positive association weakened beyond about 25 days.

Do expressions of unquestioning affiliation in language on the first day of posting predict how long people would stay active in the respective groups? For each member, a new unquestioning affiliation score was computed based on only the comments they made on their day of joining. Long-term members of the groups expressed more unquestioning affiliation even in their first day on the group (*The_Donald* : *d_>__40_ _vs.__1__–__5_* = 0.21*_;_ hillaryclinton: d_>__40_ _vs__.1__–__5_* = 0.14). To illustrate, in *The_Donald*, members with joining-day unquestioning affiliation scores in the top third of the sample remained in the group for about 6.8 days more than those in the bottom third.

*Robustness checks*. The association between unquestioning affiliation and retention remained robust when controlling for the user's Reddit activity outside of the *The_Donald* and *hillaryclinton* communities. These patterns held for total number of comments posted, number of active days, and the number of communities they posted in, both before and after they joined the *The_Donald* or *hillaryclinton* communities (more details in SOM-IX). The findings remained robust when membership duration was analyzed (a) as a continuous variable, (b) after log-transformation, and (c) using hazard models. As shown in Figure S4 (Supplementary Material) of SOM-XI, the probability of staying active in the community remained higher for people whose language on day of joining received high unquestioning affiliation scores.

Overall, the unquestioning affiliation index, when applied to the daily conversations of the 2 subreddits reflected meaningful individual differences in group identity strength. Specifically, the language of long-term members indicated higher levels of unquestioning affiliation, even on their day of joining the group. In other words, our language index was able to identify members with strong group identities based on only the language they used on their day of joining.

## Study 3b: Does the Unquestioning Affiliation Index Reflect Meaningful Temporal Changes Associated With Members’ Joining and Leaving of Groups?

The longer that people spend in a group interacting with other members, the more their group identity should strengthen. Similarly, their group identity should weaken prior to their departure from the group. The unquestioning affiliation index applied to conversations was expected to reflect this hypothesized pattern.

### Methods

The *The_Donald* and *hillaryclinton* datasets were used to track within-person shifts. As in the previous analysis, LIWC scores were standardized within each date. Each member's daily contributions to the group were then chronologically ordered and numbered. To ensure that there were sufficient data points to observe changes over time, the analysis included only people who posted on 10 or more days.

A total of 2 types of temporal shifts were examined corresponding to members’ initial days after joining a group and final days before leaving a group. As in the previous analyses, the sample was divided into 3 arbitrary categories based on membership duration (i.e. those who stayed for 10–19 days, for 20–39 days, and for 40 or more days). To visualize within-person temporal effects for each category that are not driven by between-person differences, the analysis included texts posted on a user's *i*th active day since joining the group and *j*th active day before leaving the group only if all members of that category posted on those days. For the 10–19 days category, only members’ first 5 active days and last 5 active days in the group were included, because all members in the category posted on at least 10 days; for the 20–39 category, members’ first 10 active days and last 10 active days were included. For the 40+ days category, the first 10 and last 30 days were included. The exclusion criteria used in the previous analysis were followed. The final analyses included texts from the initial days of 49,679 *The_Donald* members and 4,289 *hillaryclinton* members and the final days of 49,337 *The_Donald* members and 4,259 *hillaryclinton* members (see SOM-XII including Table S6 (Supplementary Material) for more details).

### Results

Separate mixed effects models were used to examine change in expressions of unquestioning affiliation over members’ (a) initial days after joining and (b) final days before leaving. In all models, comments’ chronological order entered as a fixed effect and intercepts were included as a random effect to account for idiosyncratic variation in individuals’ baselines.

**Fig. 2.**
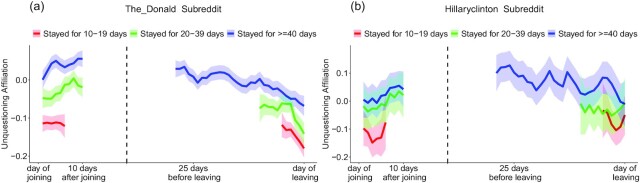
(a) and (b). Change in unquestioning affiliation expressed in natural language after joining and before leaving *The_Donald* (2a) and *hillaryclinton* (2b). The *y*-axis shows 3 days rolling means of the unquestioning affiliation score. The graphs indicate that linguistic expressions of unquestioning affiliation generally increase after joining a group and drop before leaving the group. Error bands represent confidence intervals.

First, as depicted by the left slopes of Fig. 2(a) and (b), members’ unquestioning affiliation increased in a linear manner in their first few days in the group (*The_Donald*: *b* = 0.010, *t*(198,841.6) = 8.04, and *R*^2^_m_ = 0.03%; *hillaryclinton*: *b* = 0.01, *t*(22,343.6) = 3.36, and *R*^2^_m_ = 0.05%; *R*^2^_m_ refers to marginal *R*^2^ or variance explained by the model's fixed effects. In Table S7 (Supplementary Material) of the SOM, we report both marginal and conditional *R*^2^). Another random intercepts HLM model examining unquestioning affiliation in members’ last days in the group revealed a significant negative slope indicating a drop in linguistic expressions of unquestioning affiliation as people approached their day of departure from the group (*The_Donald*: *b* = −0.005, *t*(274,803.6.5) = −14.6, and *R*^2^_m_ = 0.07%; *hillaryclinton* : *b* = −0.004, *t*(31,390.3) = −4.87, and *R*^2^_m_ = 0.07%).

*Robustness checks*. We also tested alternate models in which the date of posting was entered as a fixed effect instead of standardizing the unquestioning affiliation index within each date, and the effects remained robust. These findings along with marginal and conditional *R*^2^ values are reported in SOM-XII (also see Figure S7, Supplementary Material). The effects were robust to controlling for users’ Reddit activity before joining *The_Donald* and *hillaryclinton* communities.

To summarize, across the 2 political groups and a replication sample presented in SOM-XIII, language markers of group identity strength, measured as expressions of high affiliation and low questioning, increased over time after people joined the group and decreased before leaving. This analysis is 1 of the first to show that group identity can change even over short periods of time. The patterns detected in *The_Donald* appear more robust than the ones in *hillaryclinton*, which is likely because of the considerably smaller sample from *hillaryclinton*. It is notable that these patterns were captured even though the dates of joining and leaving were vastly different across the sample, differing by months or even years, which may explain the small effects.

## General Discussion

A total of 3 studies examining a range of groups in diverse contexts demonstrated that people's language contains measurable traces of their group identities and, more importantly, that such markers predict important behavioral outcomes and reflect ongoing dynamic processes. The analyses uncovered 2 language-based markers of group identity strength. First, people with strong group identities used more affiliation words, presumably reflecting the fundamental notion that group identities bring together collectives and forge strong intragroup relationships (3, 30). Second, people with strong identities used fewer cognitive processing words—which are typically used to work through or question unresolved issues—suggesting that strong group identity stills the mind when it comes to group-related issues. This finding aligns with the growing evidence that extreme identities and ideologies are linked with higher levels of certainty (20, 21, 31, 32). Notably, 2 central markers of identity—affiliation and questioning—reflected not just individual differences in group identity strength but also change over time. The longer people spent in identity-based groups, the more affiliation they expressed and the less they questioned.

Using the affiliation and cognitive processing dimensions, we formulated a language-based unquestioning affiliation index that predicted not only self-reported group identity but also offline and online behavioral outcomes. Study 2 found links between students’ language and an important real-world outcome that is difficult to predict: retention in college. In Study 3, unquestioning affiliation measured from naturalistic conversations in large online political communities predicted how long a member would remain with the group, and reflected temporal psychological changes that individuals undergo after joining and before leaving groups.

The current project is inspired by and contributes to the rich literature on social and group identity. Historically, group identity has been studied primarily using individuals’ self-reported feelings and perceptions, with relatively few attempts to capture how group identity is expressed in natural language. It is noteworthy that language is, by definition, a social behavior. People's language within group contexts tells us not only about their psychological states but also about how they are conveying their identities to each other during their interactions. The current study highlights the importance of 2 such facets of group identity—affiliation and certainty—which people explicitly or implicitly expressed in their conversations. Our evidence that people communicate the strength of their identities in their utterances to fellow group members raises the possibility that group members detect such signals and use this information to shape their perceptions of, and future interactions with, the speaker. These processes may operate alongside previously identified tendencies of language alignment with group members (10, 11). Future research should investigate social dynamics of language expressions of identity and also explore other features of identity that get conveyed in natural language.

In addition to the theoretical findings, the language-based approach developed in this research has important methodological implications. First, as shown in Study 3b, the language-based approach is unique in its ability to capture day-to-day variations in identity strength. To our knowledge, this is the first study to show that group identity strength can fluctuate over short periods of time and as a function of ordinary events like joining and leaving of groups. Second, this work goes beyond previous research on the online behaviors of strongly identified individuals (33, 34) by opening the possibility of studying identity processes in emerging identity-based online groups. This approach can be especially useful in studying extreme groups that have been linked with contemporary social problems (e.g. rising partisan animosity; Marchal, 2020), and which would be otherwise hard to reach (e.g. the QAnon community; (35).

The current findings have potential applicability across several domains. The language-based index could be used to track identities in a range of within-group contexts—for example, conversations within extremist groups, politicians’ language within ingroup contexts, and so on. Although this work focused on within-group contexts, the general method suggests that it may also be possible to formulate ways of tracking group identity in conversations with outgroup members. While the current goal of measuring group identity required us to fold together the 2 focal dimensions—affiliation and cognitive processing, future work may track them separately. Future research will also undoubtedly investigate these and other language dimensions to explore different facets of identity. Further, in line with the social identity tradition, we considered identity processes that broadly apply across different types of groups and categories—national, political, religious, and educational groups—without differentiating between them. But researchers with a focus on prediction, as opposed to theory advancement, or who want to focus on specific groups may devise more nuanced algorithms by accounting for different types of groups or contexts, for instance, by assigning context-specific weights to each variable.

Data from social media groups, like all naturalistic data, are noisy, leading to small effects (36, 37). This is evidenced by the smaller effect sizes in the Reddit analysis in Study 3 relative to the effects detected via Study 1’s surveys. Effect sizes from surveys and lab studies are usually inflated because these methods eliminate noise that is characteristic of our complicated world. Given the large sample sizes in big data studies, we can be sure that the detected effects, even if small, reliably capture true psychological phenomena (38) (see SOM-IX for a detailed discussion). Further, the analysis of such large corpora raises some important ethical questions given the potential privacy risks to users. Researchers using digital data should adhere to applicable legal and ethical guidelines (39) to provide protections to users of these websites.

The current research is not without limitations. A central issue in language analysis is that the meaning of linguistic expressions almost always depends on context. The current research focused on identity-based groups, wherein members feel committed to a shared belief or entity, but the findings may not extend to groups whose purpose is to question various issues (e.g. a community of scientists). Further, there might be some situations (e.g. under threat), wherein concerns for the group might induce the members with the strongest identities to exhibit highest levels of questioning, rendering our language-based metric less accurate in such instances. Our exploration into language content was preliminary (see Tables S2, S4, S8–10, and Figures S12 and S13 [Supplementary Material] in the SOM), but future research might conduct more comprehensive analyses. Similarly, we focused on common language markers that surfaced across contexts—when people wrote about a group (Studies 1 and 2) or engaged in naturalistic conversations within the group (Study 3). Future work could take a more context-aware approach. Furthermore, while we focused on intragroup conversations, future research might examine how identity processes manifest in intergroup interactions.

The current research offers a new approach to capturing group identity strength that takes advantage of the abundant information that conversations within groups bear about the psychological processes that undergird group dynamics. While this work provides only a glimpse into the types of research possible via naturalistic observation of groups’ language, this arena is rife with research questions. What is clear is that the language approach combined with a focus on online communities has the potential for studying group dynamics on a vastly different scale and temporal horizon than has been possible so far.

## Authors' Contributions

A.A. & J.W.P. designed research; A.A. performed research; A.A. & J.W.P. planned analyses; A.A. analyzed data; and A.A. & J.W.P. wrote the paper.

## Supplementary Material

pgac022_Supplemental_File
