# Correction to: Tracking group identity through natural language within groups

**DOI:** 10.1093/pnasnexus/pgad457

**Published:** 2024-01-30

This is a correction to: Ashwini Ashokkumar, James W Pennebaker, Tracking group identity through natural language within groups, *PNAS Nexus*, Volume 1, Issue 2, May 2022, pgac022, https://doi.org/10.1093/pnasnexus/pgac022

During a retroactive audit conducted by *PNAS Nexus*, it was discovered that this paper was missing a statement acknowledging compliance with the *PNAS Nexus* Human and Animal Participants and Clinical Trials policy:

The University of Texas IRB approved all of our studies. We collected original data in studies 1a-1c, and study 2, and these studies sought and obtained consent from participants. In study 2, which was an archival study, students who did not consent for their data to be used for research were excluded. Study 3 used publicly available, archival data from social media, and so informed consent is not relevant.

This error has been corrected in the original article.

