# Supplementary material for: Tracking group identity through natural language within groups
Source: PNAS Nexus. 2022 Jun 24;1(2):pgac022. doi: 10.1093/pnasnexus/pgac022 (PMC9229362; doi:10.1093/pnasnexus/pgac022)
Supplement: pgac022_Supplemental_File [file pgac022_supplemental_file.docx]

Tracking Group Identity Through Natural Language Within Groups

**Supplemental Online Materials**

**Table of Contents**

[I. Open Data Access 2](#_Toc93268105)

[II. Ethics 2](#_Toc93268106)

[III. Note on Theoretical Framework 3](#_Toc93268107)

[IV. Note on LIWC and Dictionaries 4](#_Toc93268108)

[V. Studies 1a-1c Additional Details on Samples, Measures, & Analysis 5](#_Toc93268109)

[VI. Studies 1a-c and 2 Analysis of Additional LIWC Categories 6](#_Toc93268110)

[VII. Study 2 Additional Details on Samples and Measures 7](#_Toc93268111)

[VIII. Study 2 Additional Analyses 8](#_Toc93268112)

[IX. Study 3 Notes on Effect Sizes and Controls 8](#_Toc93268113)

[X. Study 3 Exclusions & Robustness Checks using Alternate Exclusion Criteria 9](#_Toc93268114)

[XI. Study 3a Additional Methodological Notes and Robustness Check Analyses 10](#_Toc93268115)

[XII. Study 3b Additional Methodological Notes and Robustness Check Analyses 14](#_Toc93268116)

[XIII. Study 3 Replication in a sample of Bernie Sanders Supporters 18](#_Toc93268117)

[XIV. We vs. Other Affiliation Words 19](#_Toc93268118)

[XV. Study 3 Analysis of Topics 21](#_Toc93268119)

[References 23](#_Toc93268120)

## Open Data Access

All data, code and the LIWC dictionaries used have been made publicly available at <https://osf.io/dwkec/>

**Studies 1a - 1c.** All variables including participants’ essays and relevant LIWC scores have been made available.

**Study 2.** The shared dataset includes all self-reported measures and academic records including self-reported fusion, demographics, enrollment records, and SAT scores. We are unable to share students’ essays to protect their identities. Instead, the dataset includes the relevant LIWC scores. If researchers would like to reanalyze the actual text, they can contact the authors.

**Study 3a - 3c.** Datasets corresponding to the three focal subreddits — *The_Donald*, *hillaryclinton*, and *SandersForPresident* — have been made publicly available. In order to protect users’ identities, complying with Reddit API terms and conditions, we do not share the text of the comments. The public dataset instead includes the relevant LIWC variables for each text. Other metadata computed from the dataset such as members’ membership duration, date of first post and last post, and so on are also included in the dataset. Note that user handles have been replaced with random IDs, and the IDs are unique within each dataset (not across datasets). Researchers affiliated with academic institutions who are interested in analyzing the comments should contact the corresponding author. The datasets will be shared after the proposed research is approved by the researcher’s Institutional Review Board.

## Ethics

**Studies 1a - 1c.** This study was approved by the authors’ institution’s Institutional Review Board.

**Study 2.** The archival analysis was approved by the Institutional Review Board of the authors’ institution. Students in the class were informed at the beginning of the semester that all information provided by them as part of class assignments would be archived and used for research in later semesters. Consent was sought to use their anonymized responses for research, and students who did not provide consent were not included in the analyses. Except for exams and required writing assignments, all surveys, class discussions, and writing samples associated with class demonstrations were optional. In an online survey, students acknowledged receiving this information along with the option of having all their data deleted at the end of the semester. Student records maintained by the university were accessed to procure information regarding students’ enrollment in future semesters. Once the enrollment data were obtained, the dataset was anonymized by pairing student IDs with randomly generated IDs and using only the random IDs in the datasets. By maintaining de-identified datasets in this manner, the data collection and analysis process followed FERPA regulations.

**Study 3**. This Reddit study was reviewed by the authors’ institution’s Institutional Review Board and deemed to be an exempt study. This was because the Reddit data analyzed is completely public and viewable by anyone. To further mitigate privacy concerns, the Reddit dataset that has been made publicly available includes neither people’s user handles nor the comments they posted. Only the LIWC variables corresponding to users’ comments are made publicly available. The authors are willing to share user handles and texts to university researchers with clearance from their university’s IRB.

## Note on Theoretical Framework

Social psychologists have conceptualized and examined social identity in several ways. For example, social identity theorists have produced a large body of evidence showing that people’s identities associated with the groups they belong to influence their cognition and behavior [(Tajfel & Turner, 1979)](https://www.zotero.org/google-docs/?broken=2mOUvX). Identity fusion theory emphasized a more extreme form of connection with groups that predicts extreme pro-group behaviors [(Swann et al., 2014)](https://www.zotero.org/google-docs/?broken=aZz6e3). Subfields within psychology and organizational behavior have focused on other constructs such as organizational commitment [(Steers, 1977)](https://www.zotero.org/google-docs/?broken=VM9wwu). These constructs, as measured by self-reports, are typically positively correlated with each other (.6 < *r*s < .9), but they use carefully worded survey items to capture the nuanced distinctions between these related constructs. Language is a relatively blunt instrument that is best for capturing broader psychological states as opposed to nuanced theoretical distinctions. The current research sought to identify the linguistic markers of group identity strength more broadly.

Surveying the literature, several facets of group identity strength were identified.

1. ***Social and socio-cognitive facets:***
   - ***Categorization*.** According to self-categorization theory, identity operates at multiple levels (e.g., personal and social identity) that have a ‘functional antagonism’ between them such that as one level becomes more salient the other levels become less so (Turner, 1994). When a group is salient or, for people who strongly identify with a group, a shift may occur from their personal to social identity, which could arguably be captured in language as a shift from I-words (first-person singular pronouns) to we-words (first-person plural pronouns). Self-categorization occurs when people perceive a shared essence or homogeneity between themselves and their group and a perception of distinctiveness relative to an outgroup (for a review, see Hornsey, 2008).
   - ***Ingroup commitment and intra-group ties.*** Several approaches emphasize the link between social identity and having a psychological bond with, and commitment to, fellow in-group members (for a review, see Leach et al., 2008). In the same vein, identity fusion theory is centered on relational ties between group members (Swann et al., 2014). This work generally suggests that group members with strong identities should feel a sense of belonging and psychological attachment to their group. This may be expressed in language using increased references to one’s collective self (e.g., we words) and other words indicating affiliation to others *(e.g., help, love, together).*
2. ***Cognitive facet–Uncertainty reduction******.*** Another classic theory in the social identity tradition is Hogg’s uncertainty-identity theory, which argues that identification is underpinned by one’s need to reduce their subjective uncertainty about what to say, do, think, or feel (Hogg, 2007). A reduction in feelings of uncertainty could provide social meaning. A drop in uncertainty need not necessarily manifest as an increase in explicit linguistic expressions of conviction or certitude. Reduction in uncertainty is better captured as a drop in language that has previously been identified as characteristic of uncertainty. Cognitive processing words, which are typically used to work through issues during times of confusion and uncertainty (Boyd et al., 2020; Seraj et al., 2021), may drop as a function of strong group identity.
3. ***Emotional facet:***
   - ***Positive self-esteem and positive affect****.* Group identification is associated with positive feelings about the group and one’s membership in it (Tajfel, 1978). This may manifest as greater positive emotion and lower negative emotion in group-related language.
   - ***Group-based emotions.*** According to intergroup emotions theory (Mackie & Smith, 2018), people’s emotional experiences are shaped by their group membership. That is, group members’ emotions should align with their group’s affective norm regardless of the emotion’s valence. Some groups may tend to experience and express more positive, than negative, emotions. But in other groups, for example, hate groups, negative emotions may be more normative. Testing this proposition would require using measures based on language similarity or word embeddings, which is beyond the scope of this paper.
   - Aligned with the theoretical discussion in the above paragraphs, in Studies 1a-1c and 2, people with strong self-reported identities expressed more positive emotion and less negative emotion while talking about their group (see Figure S1 later in this document). This likely indicates the positive associations that strongly identified members have with their group. In contrast, when members of *The_Donald* engaged in natural conversations within their group, we found different patterns: Long-term members of *The*_*Donald* expressed higher levels of positive and negative emotions on their first day in the group. In *hillaryclinton*, we found an association between membership duration and positive, but not negative, emotions. We also did not find consistent temporal changes across the groups. These preliminary analyses suggest that strong identity is associated with positive emotion when writing *about* a group in a survey setting, but emotional expressions in ingroup conversations may depend on the specific group and situation where emotion is expressed. Determining when social identity leads to greater positivity and when people’s emotions depend on group norms is an important question, which we leave for future research.
4. ***Behavioral facet– high engagement in performative tasks******.*** People with strong group identities are most motivated to engage in group-related performative behaviors that explicitly advertise their group identity (Klein et al., 2007). They may then indirectly express their group identity via higher levels of engagement in a writing task about a group or by talking more with fellow group members, which can be captured by simply counting the number of words spoken.

## Note on LIWC and Dictionaries

The affiliation and cognitive processing dictionaries are part of LIWC2015. LIWC is a dictionary-based text analysis program that can be used to capture psychological states from text. LIWC2015 contains a number of dictionaries (e.g., affiliation, risk, death), each containing lists of words identified and validated via an iterative, multi-step process (see Pennebaker, Boyd, Jordan, & Blackburn, 2015 for more details on the process and for psychometric information). When LIWC is applied to a text, it determines the percentage of words in the text that belong to each dictionary. This score tells us the degree to which the text focuses on the corresponding concept. Because the score represents percentages, short texts do not provide reliable estimates. For instance, a comment with just one word would be scored as 100% on the corresponding category. For this reason, short texts are typically not analyzed. The list of words in the affiliation and cognitive processing dictionary are provided [here](https://osf.io/dwkec/?view_only=2b2533e512944f529f03d233f2b635f5).

## Studies 1a-1c Additional Details on Samples, Measures, & Analysis

**Sample & Exclusions.** MTurkers with a HIT approval rating of at least 95% were recruited. Participants were excluded if they did not follow survey instructions (N = 7 in Study 1a; N = 1 in Study 2b) or if they failed any attention check question embedded in the survey (N = 4 in Study 1a; N = 6 in Study 1c). Participants in Study 1b who reported being agnostic or atheistic wrote about their relationship with their respective belief system (agnosticism or atheism). Participants in Study 1c who had never attended college were not allowed to participate.

**Demographics.** Study 1 (56.7% female; *M_age_ =* 39.02*; SD_age_ =* 13.11; 76.3% White), Study 1b (53.7% female; *M_age_ =* 36.89*; SD_age_ =* 12.38; 76.2% White), and Study 1c (49.2% female; *M_age_ =* 34.65*; SD_age_ =* 10.88; 79.4% White).

**Measures.** On average, participants wrote 169.3 (1a), 138.8 (1b), and 160.6 (1c) words**.** Means and standard deviations of self-reported and linguistic measures are reported in Table S1. Reliability indices are as follows:

- - - fusion: .86 < *α*s < .94
    - pro-group action: .78 < αs < .84
    - LIWC affiliation: .40 (Cronbach), .80 (standardized Cronbach)
    - LIWC cognitive processing: .65 (Cronbach), .92 (standardized Cronbach)

**Table S1.** Descriptive statistics for self-reported and linguistic measures in Study 1a-1c

|  | Study 1a:  U.S.A  M (SD) | Study 1b:  Religion  M (SD) | Study 1c:  College  M (SD) |
| --- | --- | --- | --- |
| Self-reported fusion | 4.59 (1.53) | 4.72 (1.57) | 3.90 (1.66) |
| Self-reported pro-group behavior | 3.79 (1.67) | 3.85 (1.40) | 3.19 (1.35) |
| LIWC Affiliation | 4.00 (2.74) | 3.38 (2.94) | 2.32 (1.81) |
| LIWC Cognitive Processing | 13.55 (4.39) | 15.54 (5.49) | 12.60 (3.85) |

In all three studies, participants provided information about the emotions they experienced during the writing task and their opinions on the group. They also provided demographic information including gender, age, ethnicity, political orientation (Study 1a and 1b), education (Study 1a) and income (Study 1c). Study materials have been made publicly available.

**Unquestioning affiliation**. Table S2 presents sample responses with low vs. high unquestioning affiliation scores. Participants whose texts received high scores tended to see the group as an important part of their lives, expressed a sense of solidarity with the group and spoke about their connection with other group members or their group’s values. On the other hand, people whose writing received low scores either explicitly expressed their lack of connection or were still making sense of what the group meant to them. Taken together, these examples provide preliminary evidence for face validity for the developed metric of group identity strength.

**Table S2.** Excerpts from essays with low & high unquestioning affiliation scores (Studies 1a-1c)

|  | **Example responses** |
| --- | --- |
| **High**  **unquestioning affiliation**  **score** | - “America our nation is the best country in this world... America has been our image to this world, we are a nation that is the model for this world”. - “I love my college, it’s like my second home” - “I love god. it's a personal relationship. He died for me and I will give my all for God. I attend church and regularly commune with his people.” - “I feel unending support and love from them. I enjoy being there for my church family and volunteering as well. It gives me a sense of fellowship and giving to others.” |
| **Low unquestioning affiliation**  **score** | - “I don't really feel a deep connection with America as a whole” - “I want to believe...I'm just not sure if I believe. I am so unsure...” - “(I) do not know all the answers. I do not know if one religion or another is more right or wrong than another” - “ I wanted to transfer out of XXXX at first because it wasn't the right fit. I never ended up doing that and I always wonder what my life would have been like if I chose a different school.” - “I felt a little lost but I think that was as much my own fault as anything. Part of feeling lost is not really knowing what to do to not feel lost!” |

**Additional statistics.** Table S3 reports statistics from models examining the link between self-reported identity strength and our language index of unquestioning affiliation while controlling for demographics. Providing evidence for divergent validity, no demographic variables were consistently associated with unquestioning affiliation.

**Table S3.** Anova statistics for demographic effects on unquestioning affiliation scores

|  | Study 1a  (N = 247)  *F* | Study 1b  (N = 372)  *F* | Study 1c  (N = 250)  *F* |
| --- | --- | --- | --- |
| Self-reported Identity Fusion | 9.76** | 16.82*** | 14.74*** |
| Gender | .03 | 2.36^†^ | .96 |
| Age | .49 | 3.27^†^ | .07 |
| Race | 1.24 | .29 | 1.89^†^ |
| Political Orientation | 1.01 | 1.39 | - |
| Education Levels | .46 | - | - |
| College degree obtained | - | - | 1.19 |

*Note.* ** indicates *p* < .01. *** indicates *p* < .001. ^†^ indicates *p* <= .1

## Studies 1a-c and 2 Analysis of Additional LIWC Categories

Our focus on affiliation, cognitive processing, I-words, and word count was based on their conceptual roots in the literature. Nevertheless, we found some consistent associations between some other LIWC categories and self-reported measures. Fig. S1 presents a plot of correlations between a comprehensive list of LIWC categories and self-reported identity fusion in four samples. LIWC categories with an average base rate lower than 1% across the samples or which were composites calculated from other LIWC categories (e.g., Tone, Clout, Analytic, etc.) are not presented.


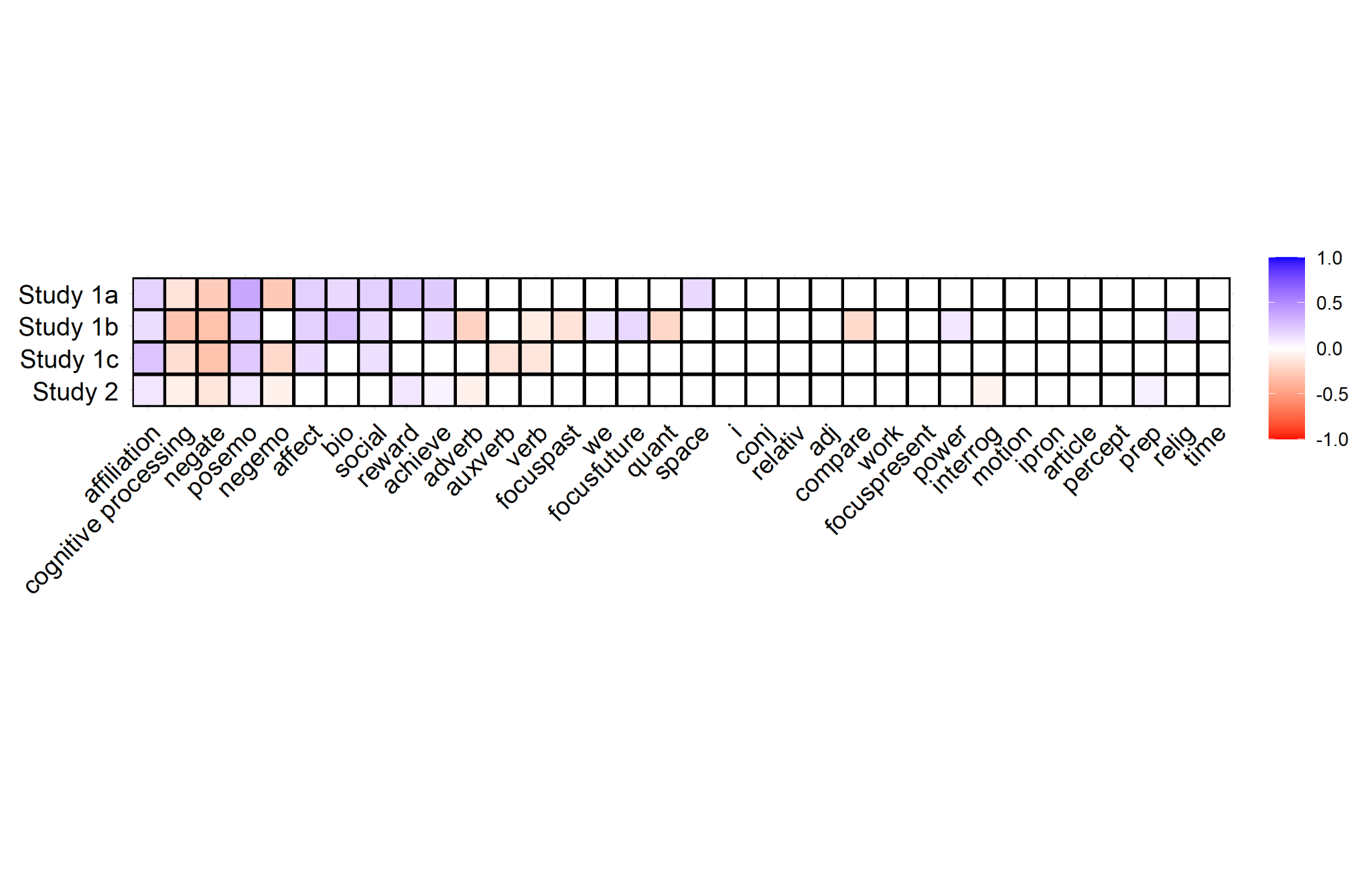


Figure S1. Correlations of LIWC categories and self-reported identity fusion in four samples. Only statistically significant cells are colored

1. Use of negation words was negatively associated with fusion across four samples. This was at least in part because most weakly fused participants began their essays by stating that they did not feel connected to the group, manifesting as negation words (not, don’t, didn’t, etc). Notably, a significant number of negation words are included in the cognitive processing dictionary, indicating overlaps between these effects.
2. A meaningful pattern can be seen with emotion categories as discussed in SOM-III.

## Study 2 Additional Details on Samples and Measures

**Sample & exclusions.** Eight students who were enrolled in either post-bachelor or graduate programs were excluded.

**Measures.** Fusion with college was measured using different scales in different semesters: Fall 2015: 5-point scale (*M* = 2.99, *SD* = 1.1); And Spring 2016: 5-point scale (*M* = 3.08, *SD* =1.14).

Sample responses are provided in Table S4. Essays that received high scores were evidently written by students who were well adjusted in college. For instance, a student whose social engagement score was among the highest said, “I have a great support system ... and I think I adjusted extremely well to moving away from my parents”. Others with high scores made references to social life in college, for example, greek life (“I love love love my sorority”) and sports (“we were able to come back and … and get a win under our belts”). In contrast, students with the lowest social engagement scores did not seem to belong at the university. The talked about feeling left out (“I only feel like an outcast... getting rejected from every social organization does that”). Another student said, “I just feel sad a lot as I'm at this school...I always question if coming here was the best option for me”. This example is striking in the author’s degree of ambivalence about whether they should stay in college.

**Table S4.** Excerpts from responses with low and high unquestioning affiliation scores (Study 2)

|  | **Example responses** |
| --- | --- |
| **High score** | - “I have a great support system ... and I think I adjusted extremely well to moving away from my parents” - “I love love love my sorority ... (I) see the faces of all my sisters and friends and know that they're probably feeling the same way I am” - “We were able to come back and … and get a win under our belts” - “Hook em Horns” |
| **Low score** | - “I only feel like an outcast... getting rejected from every social organization does that” - “I just feel sad a lot as I'm at this school...I always question if coming here was the best option for me” |

## Study 2 Additional Analyses

The findings reported in the article were not moderated by demographics such as gender, age, and ethnicity

**Self-reported fusion vs. language.** When self-reported fusion and the linguistic metric were simultaneously entered in the model, self-reported fusion (OR = 1.52, 95% CI = [1.26, 1.86], Wald *χ ^2^* =17.8, *p* < .001) out-predicted the linguistic metric (OR = 1.15, 95% CI = [1.02, 1.32], Wald *χ ^2^* =4.7, *p* = .03), which is not surprising given that the linguistic metric was based on essays in which students were not prompted to write about their connection with the university.

**Separate analyses of affiliation and questioning.** Self-reported identity fusion with university was positively associated with using words relating to affiliation, *r*(1510) = .10; *p* < .001, and negatively associated with words related to questioning, *r*(1510) = -.08; *p* = .002. We tested a logistic binomial regression to predict staying in university one year later. Students whose language indicated higher affiliation were more likely to remain in college after one year (OR = 1.43, 95% CI = [1.15, 1.80], Wald *χ ^2^* =9.9, *p* = .002), but there was no such effect of language indicating questioning (*p* = .34).

**Analysis using alternate writing prompt.** In addition to the primary dataset analyzed in this study, we had access to a different writing assignment completed by a third cohort of students (*N* = 649). Unlike in the main dataset wherein participants wrote about current thoughts and feelings, participants in this cohort wrote retrospectively about their experience of coming to college. The texts were shorter than in the main article (334 words, as opposed to 709 words in the main dataset). The unquestioning affiliation score was associated with self-reported fusion (*r*(647) = .11, *p* = .004) but not retention (OR = .97, 95% CI = [.82, 1.17], Wald *χ ^2^* = .08, *p* = .78). Perhaps writing about current, rather than past, feelings better reflect students’ current university identities.

## Study 3 Notes on Effect Sizes and Controls

**Effect sizes.** The effect sizes in the Reddit analyses are relatively small which is inherent in naturalistic conversational data from social media [(Kern et al., 2016; Matz et al., 2017)](https://www.zotero.org/google-docs/?broken=uVcTul). Noise stemming from variations in topics, events in the world, and other sources make Reddit a conservative context to test groups-related hypotheses in. Other researchers have noted that small effect sizes are particularly valuable when detected using inauspicious designs or contexts [(Cortina & Landis, 2008)](https://www.zotero.org/google-docs/?broken=JMiR4J). If it is true that the noise inherent in social media data deflates the detected effects, measures taken to reduce noise and improve reliability should improve effect sizes (see Kern et al., 2016 for a detailed discussion). One way of reducing noise is to aggregate participants’ scores over several posts. Accordingly, in Study 3a, while testing whether a member’s language was associated with how long they stayed in the group, we averaged each member’s LIWC scores across several posts prior to the analysis. On the other hand, the temporal analyses in Study 3b tracked each individual’s daily changes, which made it difficult to reduce noise via aggregation, resulting in miniscule effects. Interestingly, even in these analyses, aggregating users’ posts into time-blocks improved effect sizes. For example, when the daily scores of *The_Donald* members are aggregated into three time-blocks corresponding to their initial posts (say each user’s first 10% texts), final posts (last 10%), and the rest of their posts (80%), the variance accounted for (R^2^) increases from .04% to .17%. Ultimately, it is notable that the studies captured meaningful psychological signals despite the abundance of noise in the dataset.

**Controls.** The primary outcome in the Reddit analysis was taken to be individuals’ duration of active membership in political subreddits. This raises the possibility that long-term members, who we assumed to be strongly identified with the community, are simply active Reddit users. In other words, without accounting for individuals’ Reddit activity outside of the political communities under study, we cannot be sure that the effects are solely driven by behavior within the community; we may be simply capturing language markers of active Reddit users. To address this issue, the analyses controlled for users’ past and current Reddit activity. For each individual in the final dataset, their entire Reddit activity history outside of the focal subreddits (*The_Donald* or *hillaryclinton*) was obtained, and the following variables were computed and controlled for:

- Total number of other communities in which the user posted *before* joining the focal community (*The_Donald* or *hillaryclinton*)
- Total number of other communities in which the user posted *after* joining the focal community (*The_Donald* or *hillaryclinton*)
- Total number of comments posted in other communities *before* joining the focal community (*The_Donald* or *hillaryclinton*)
- Total number of comments posted in other communities *after* joining the focal community (*The_Donald* or *hillaryclinton*)
- Total number of active days on which the user posted in other communities *before* joining the focal community (*The_Donald* or *hillaryclinton*)
- Total number of active days on which the user posted in other communities *after* joining the focal community (*The_Donald* or *hillaryclinton*)
- The timespan between when the user joined Reddit and when they joined the focal community (*The_Donald* or *hillaryclinton*)

## Study 3 Exclusions & Robustness Checks using Alternate Exclusion Criteria

The following exclusion criteria were used to pre-process the Reddit datasets:

- - - To eliminate bots, usernames that ended with “Bot” or case variations of “_bot” and “-bot” were eliminated. Some other bots were manually identified and excluded: *AutoModerator*, *autotldr*, *TheWallGrows*, *trumpcoatbot*, *autourbanbot*, *topredditbot*, *TheWallGrowsTaller*, *TweetPoster*, *heyheyitsteytey*, *word_clouds_*, *__word_clouds__,* and *topredditbot.*
    - Comments with fewer than 25 words or over 1000 words were excluded. As mentioned on the [LIWC website](https://liwc.wpengine.com/how-it-works/), dictionary-based language analysis is less reliable with texts that are too short (Boyd, 2017). It is common practice to set minimum word count thresholds. Comments over 1000 words were excluded because these are often copy-pasted from articles or other sources. While we had a clear rationale for excluding texts based on word count, the thresholds (25 and 1000) are admittedly arbitrary. To ensure that such our findings are not specific to such arbitrary decisions, we conducted robustness checks using alternate exclusion criteria, as explained below.
    - To eliminate comments written in languages other than English, comments were included only if the LIWC dictionary contained at least 50% of their words.Only a small proportion of texts (.07-.09% of the sample) were excluded based on this criterion, evading concerns regarding loss of data. Nevertheless, as shown below, eliminating this exclusion criterion did not affect the study findings.
    - Comments with high levels of repetition were excluded to prevent unusual repetition of words (e.g., the word “we” repeated 100 times) from over-influencing the LIWC scores. Repetition within comments was measured in a rolling window manner using a program called Repeatalizer (<https://repeat.ryanb.cc/>). Different exclusion rules were applied depending on the length of the comment. Comments with 25-50 words were included only if they had repetition rate lower than 40% among single content words (e.g., “politics”), 50% among single function words (e.g., “we”), 35% among 2-gram words (strings of two words such as “identity politics”), 20% in 3-gram words (e.g., “the United States”), and 15% in 4-gram words (e.g., “United States of America”). Comments with over 50 words were included if they had repetition rate lower than 25% among single content words, 35% in single function words, 40% in 2-gram words, 25% in 3-gram words, and 20% in 4-gram words. The thresholds were identified based on sampling comments with a range of repetition scores and carefully evaluating them on the degree to which they resembled ordinary online conversation.
    - Whereas the above-described exclusion criteria for comments were applied for the LIWC analysis, all comments were included while calculating the total number of posts that a user posted.
    - ***Study 3a.*** As mentioned in the article, the samples were split into three categories of “membership duration” based on the number of days that users actively contributed to the group: Members who contributed for 1-5 days, for 6-40 days, and for 41 or more days (More details on the bucketing procedure are provided in SOM-XI). Members who posted comments on fewer than 40 days before data collection but who may have gone on to post on 41+ days after data collection would have been incorrectly categorized into the first two categories. To prevent this, only the 1-40 day posters who did not post in the last two months of the dataset, who presumably left the group, were included in the analysis.

**Robustness checks testing alternate exclusion criteria.** The main models were re-analyzed without excluding any comments based on usernames resembling bots, the percentage of words in the LIWC dictionary, or repetition within comments. The minimum word count threshold was set to 10 words to ensure that the dictionary scores are at least somewhat reliable. There was no maximum word count threshold. The final sample had 2,134,857 texts from 320,258 users in *The_Donald* and 208,183 texts from 34,246 users in *hillaryclinton.* All the findings generally remained robust. Replicating findings in Study 3a, people who expressed higher levels of unquestioning affiliation in their language remained active in their community longer (*The_Donald*: Spearman’s *ρ* = .10; *hillaryclinton*: Spearman’s *ρ* = .10). Replicating findings from Study 3b, members’ linguistic expressions of unquestioning affiliation increased in a linear manner in their first few days in the group (*The_Donald*: *b* = .006, *t*(314431.6) = 6.46**;** *hillaryclinton*: *b* = .005, *t*(29892.4) = 1.68, *p¸*= .09) and dropped as they approached their day of departure from the group (*The_Donald*: *b* = -.003, *t*(359265.4) = -12.8**;** *hillaryclinton* : *b* = -.003, *t*(37384.9) = -3.25, *p¸*= .001).

## Study 3a Additional Methodological Notes and Robustness Check Analyses

**Additional sample details and statistics**

**Table S5.** Sample details broken down by duration of membership in the group (Study 3a)

|  | Stayed for 1-5 days | Stayed for 6-40 days | Stayed for >40 days |
| --- | --- | --- | --- |
| *The_Donald* | 201,019 texts from 147,163 members | 403,610 texts from 62,269 members | 2,361,212 texts from 35,249 members |
| *hillaryclinton* | 26,918 texts from 19,287 members | 44,192 texts from 5,140 members | 113,092 texts from 1,676 members |

For the anova models reported in the article, F values are as follows. The effect of membership duration on *average* unquestioning affiliation scores: (*The_Donald*: *F*(2, 244671) = 1526.8; *hillaryclinton*: *F(*2, 26078) = 71.6). The effect of membership duration and unquestioning affiliation expressed on *members’ first day on the group*: (*The_Donald* : *F*(2, 158524) =456,; *hillaryclinton: F*(2, 20977) = 24.4).

**Bucketing procedure.** In the figures shown in the article, we treated membership duration as a categorical variable. The bucketing procedure was somewhat arbitrary but helped us visualize the data in a straightforward way. We bucketed the data because the number of days that participants remained active in the Reddit communities was heavily skewed. The majority of participants remained active for only a couple of days, and only a small proportion of participants stayed beyond about 35-40 days. The median number of days that a member in the Trump community remained active was 3 days, and mean was 23.1 days. In the Clinton supporter community, the median and mean were 2 and 10.1 days respectively. One of the ways we determined the cutoffs was by eyeballing the distribution of participants’ membership duration. As shown in the figure below, the majority of the sample left the community within 5 days of joining. The graph flattens around the 35 or 40 day mark, and so we treated people who stayed 40+ days as a separate group. Another consideration was to balance the number of individuals and texts. As described above and as shown in Table S5, the number of short-term members was high, but the number of posts for this group was low because each short-term participant commented only a couple of times. On the other hand, while only a small proportion of people stayed beyond 40 days, the number of texts from this group was high because each participant contributed several times. The 1-5, 4-39, and 40+ grouping allowed us to balance the N of posts and participants. Nevertheless, because the cutoffs were arbitrary, we conducted additional robustness checks described below.


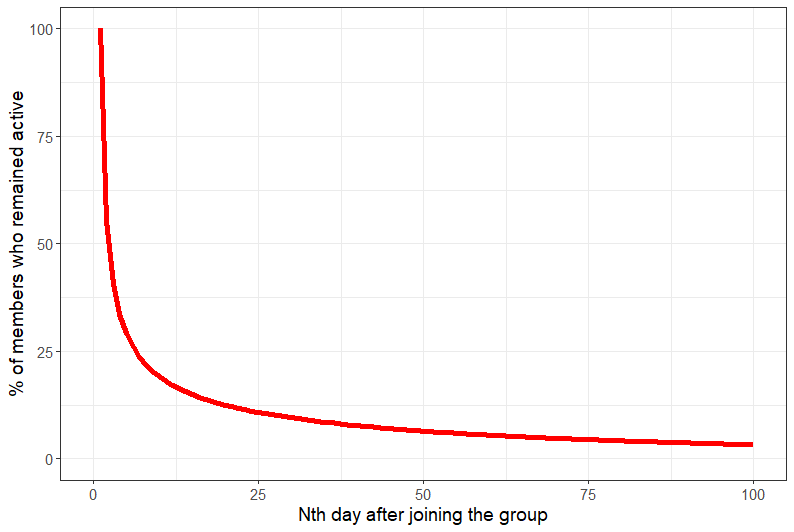


Figure S2. The percentage of members of *The_Donald* who remained active on the nth day after joining the group.

**Analysis without bucketing.** We also tested models treating membership duration as a continuous variable (i.e., without bucketing). Correlations are reported in the article. Note that log-transforming the membership duration variable to reduce skew strengthened the effect.

| **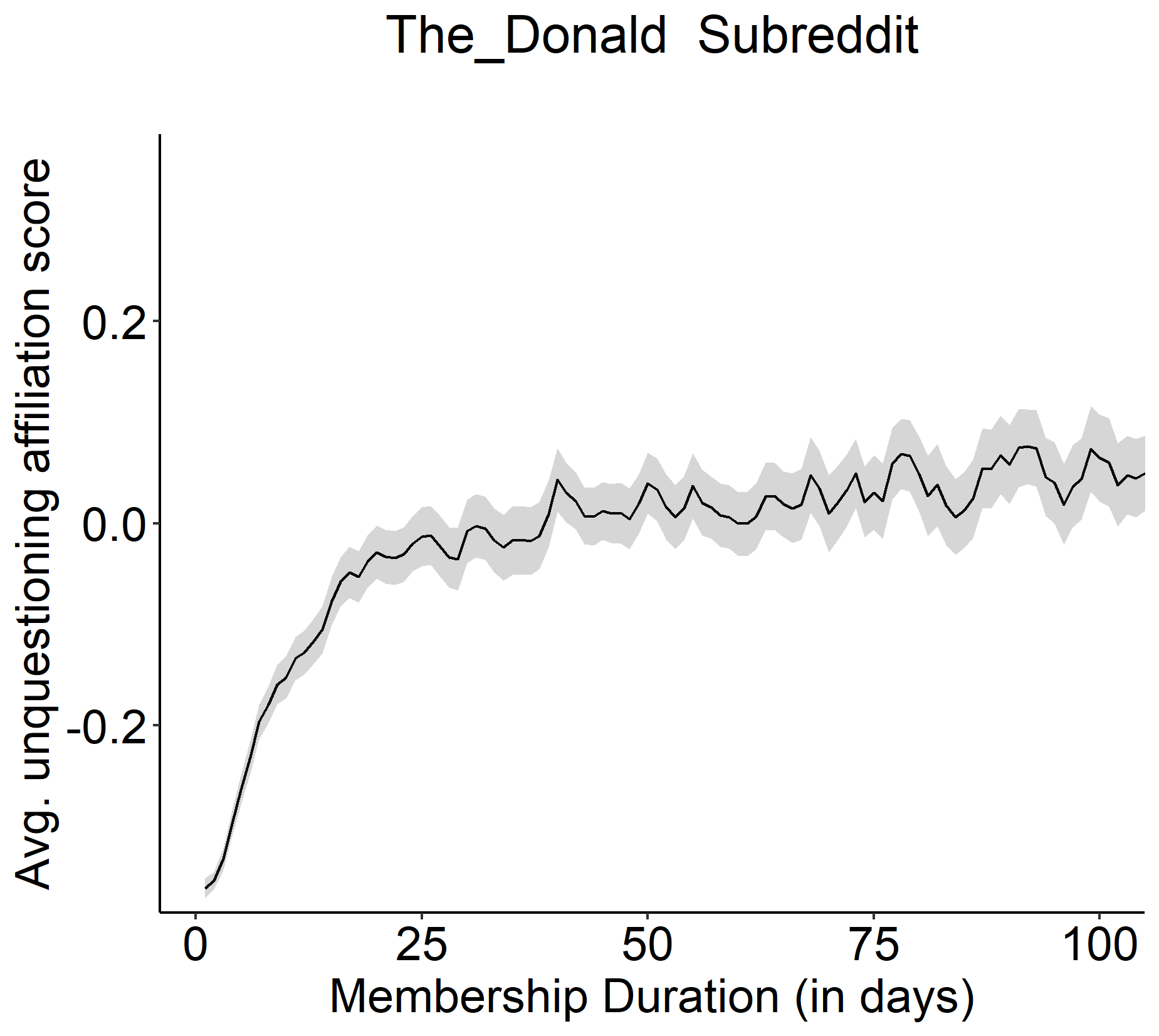** | **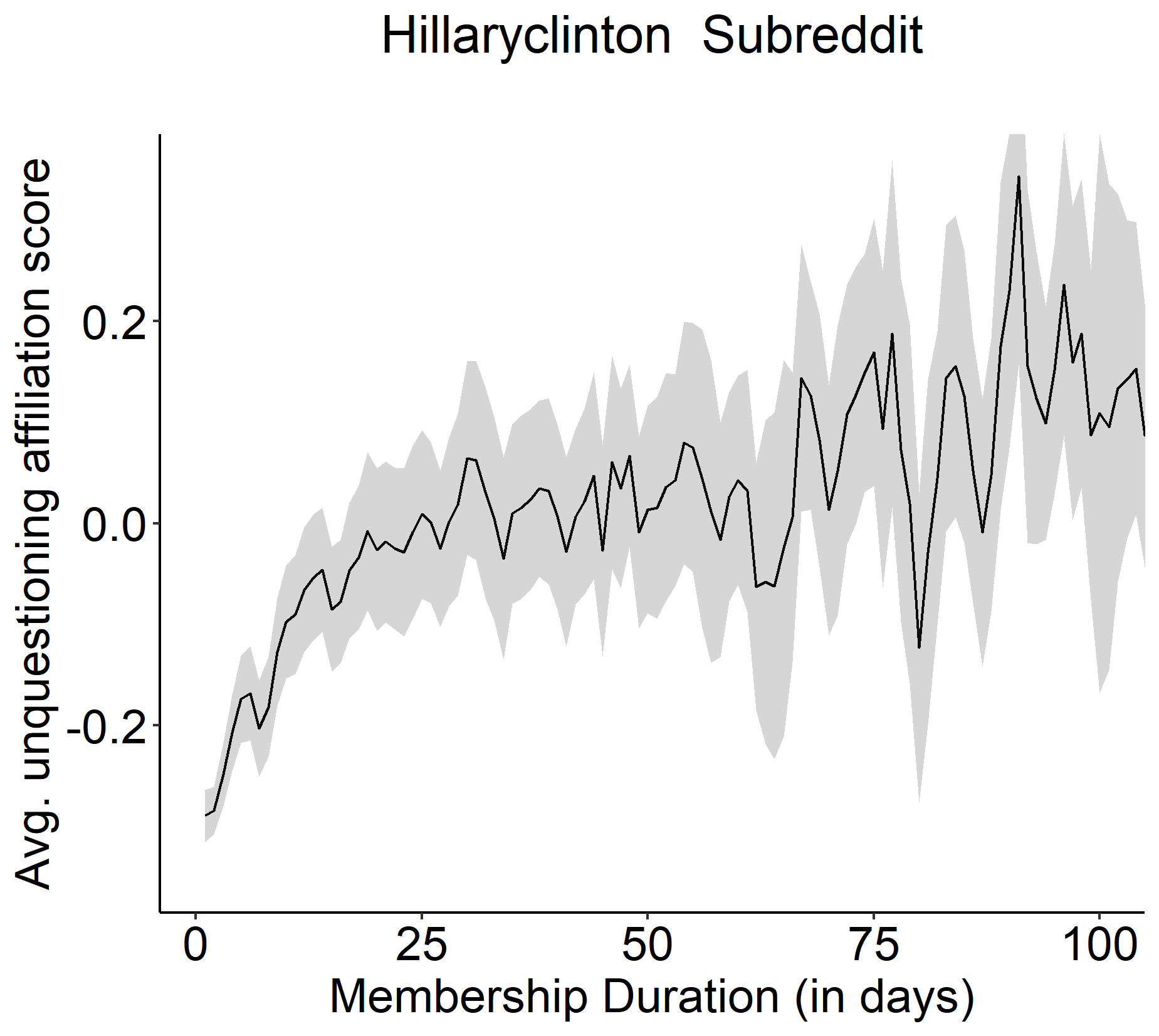** |
| --- | --- |

Figures S3a and S3b. Rolling means of unquestioning affiliation scores as a function of the number of days a member remained in The_Donald and HillaryClinton.

**Hazard model.** In addition to the anova models and correlations reported in the article, we also tested hazard models to examine the association between unquestioning affiliation on Day 1 and the probability of survival. The Cox proportional hazards regression model was used. The findings paralleled the results reported in the article: Members’ hazard or risk of leaving was lower when they used more language markers of identity (*The_Donald:* *b* = -.08, *HR* = .92, z = -31.3; *hillaryclinton:* *b* = -.06, *HR* = .94, z = -8.23). The hazard ratios (HR) suggest that a one standard deviation increase in expression of unquestioning affiliation on the first day corresponded to a reduction in hazard by 8.05% in *The_Donald* and 5.81% in *hillaryclinton.* The figures below depict the probability of survival (ie., remaining active) in the community at any point as a function of unquestioning affiliation score on their day of joining. As seen below, people whose language received higher unquestioning affiliation scores had greater chances of remaining active.

| ***The_Donald*** | ***hillaryclinton*** |
| --- | --- |
| 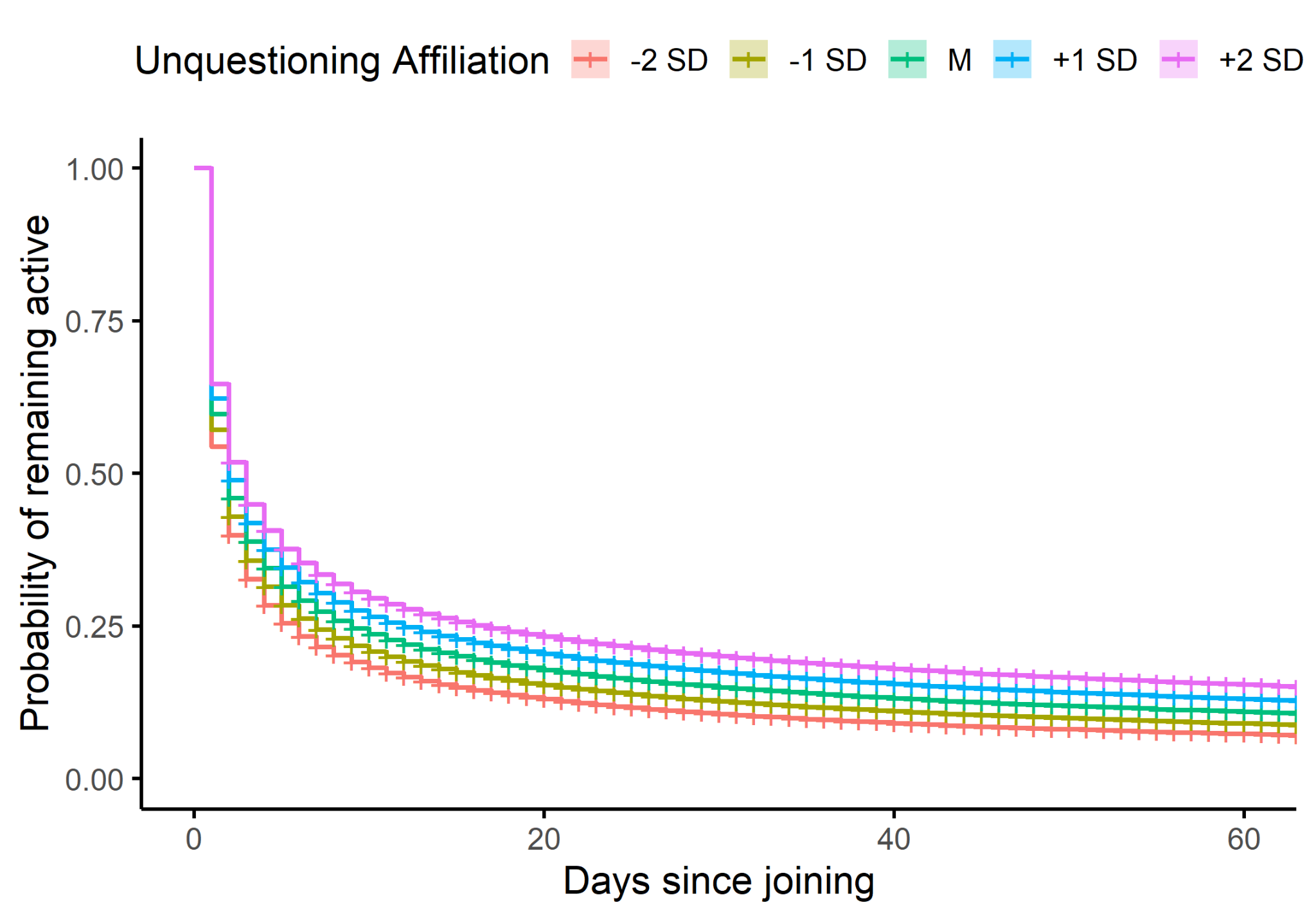 | 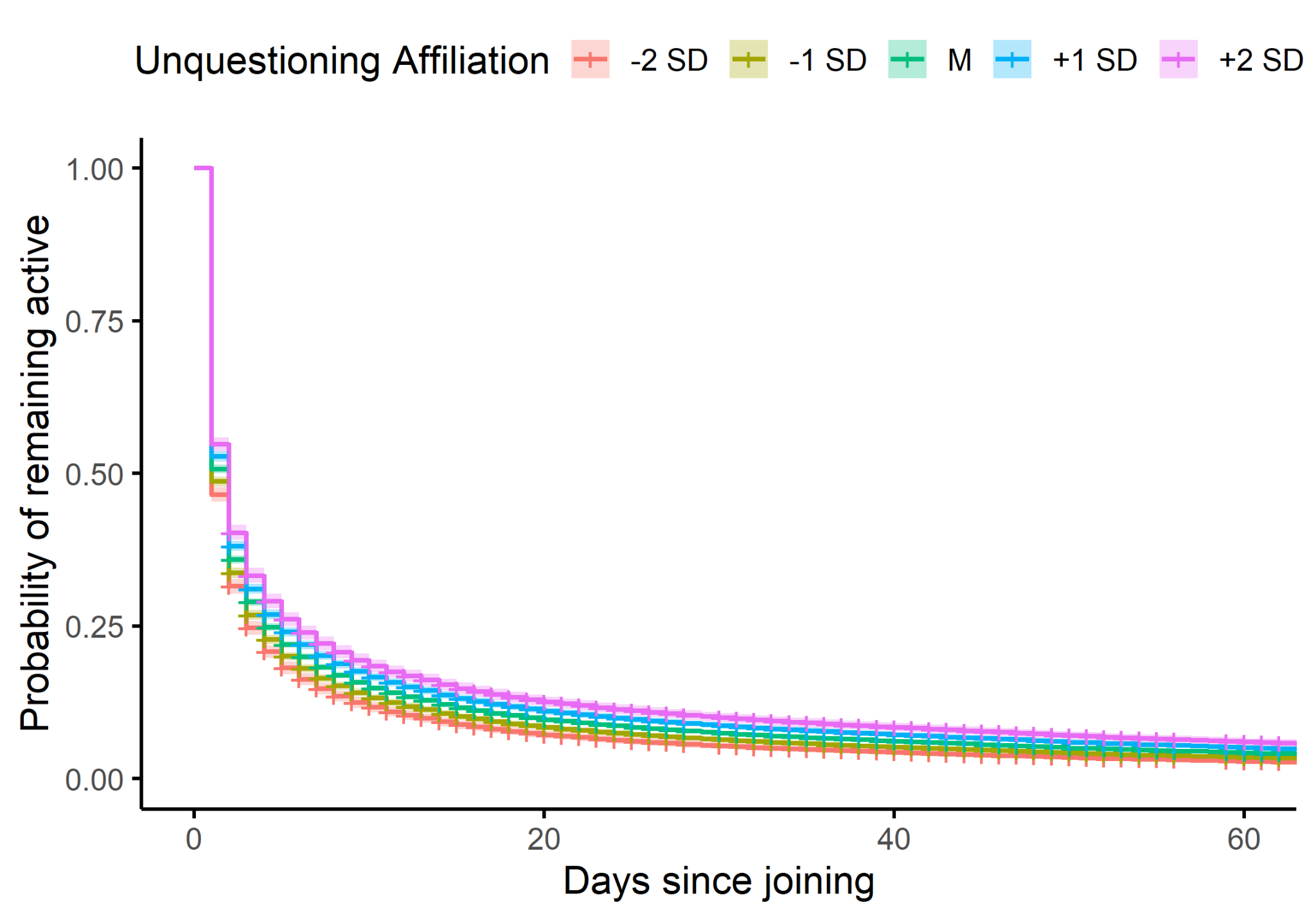 |

Figure S4a and S4b. Survival plots depicting group members’ probability of remaining active at any point as a function of the level of unquestioning affiliation they expressed on their first day

**Separate analyses of affiliation and questioning.** Separate analyses were done to determine whether the two proposed components of group identity strength⁠— affiliation and questioning ⁠— each predicted how long members remained active. The method reported in the article was used to categorize members based on how long they remained committed to the group (see Table S5 for sample sizes). Each member’s average affiliation and questioning scores across posts were computed. One-way anovas were conducted with membership duration category as independent variable and members’ average affiliation or questioning score as dependent variable. All the Reddit analyses reported below were statistically significant at *p* < .001 unless otherwise specified**.** Figures S5a and S5b reveal that members who remained active in the group for longer used more affiliation words (*The_Donald*: *F*(2, 244678) = 264, *d_>40 vs 1-5_* = .11; *hillaryclinton*: *F(*2, 26100) = 13, *d_>40 vs 1-5_* = .11). Members who remained active in the group for longer were also less questioning than the short-term members (*The_Donald*: *F(*2, 244678) = 1654, *d_>40 vs 1-5_* = -.29; *hillaryclinton*: *F(*2, 26100) = 81.9, *d_>40 vs 1-5_* = -.24; see Figures S6a and S6b).

| **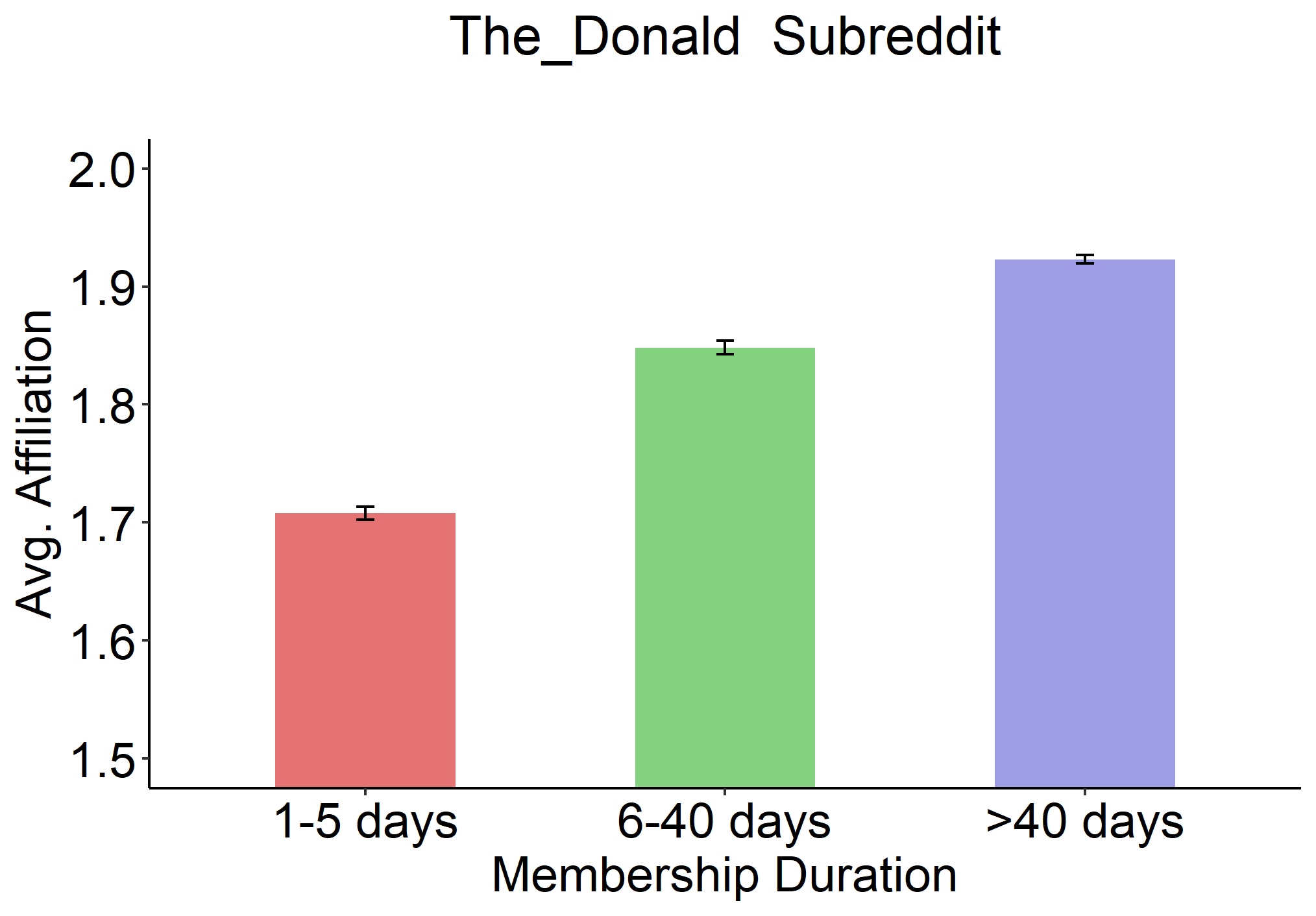** | 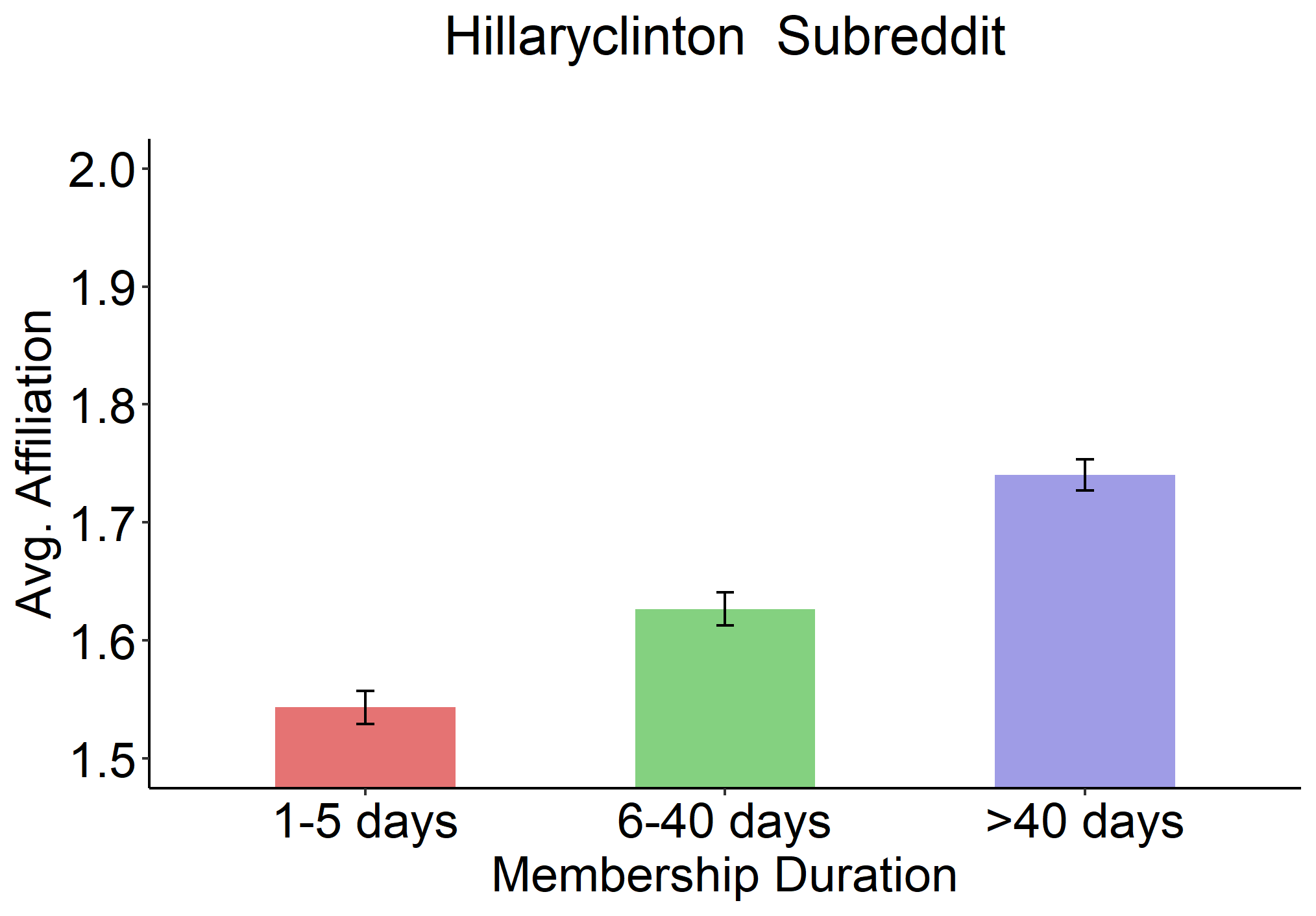 |
| --- | --- |

Figures S5a and S5b. Means of affiliation for short-, medium- and long-term members of *The_Donald* and *hillaryclinton*.

| 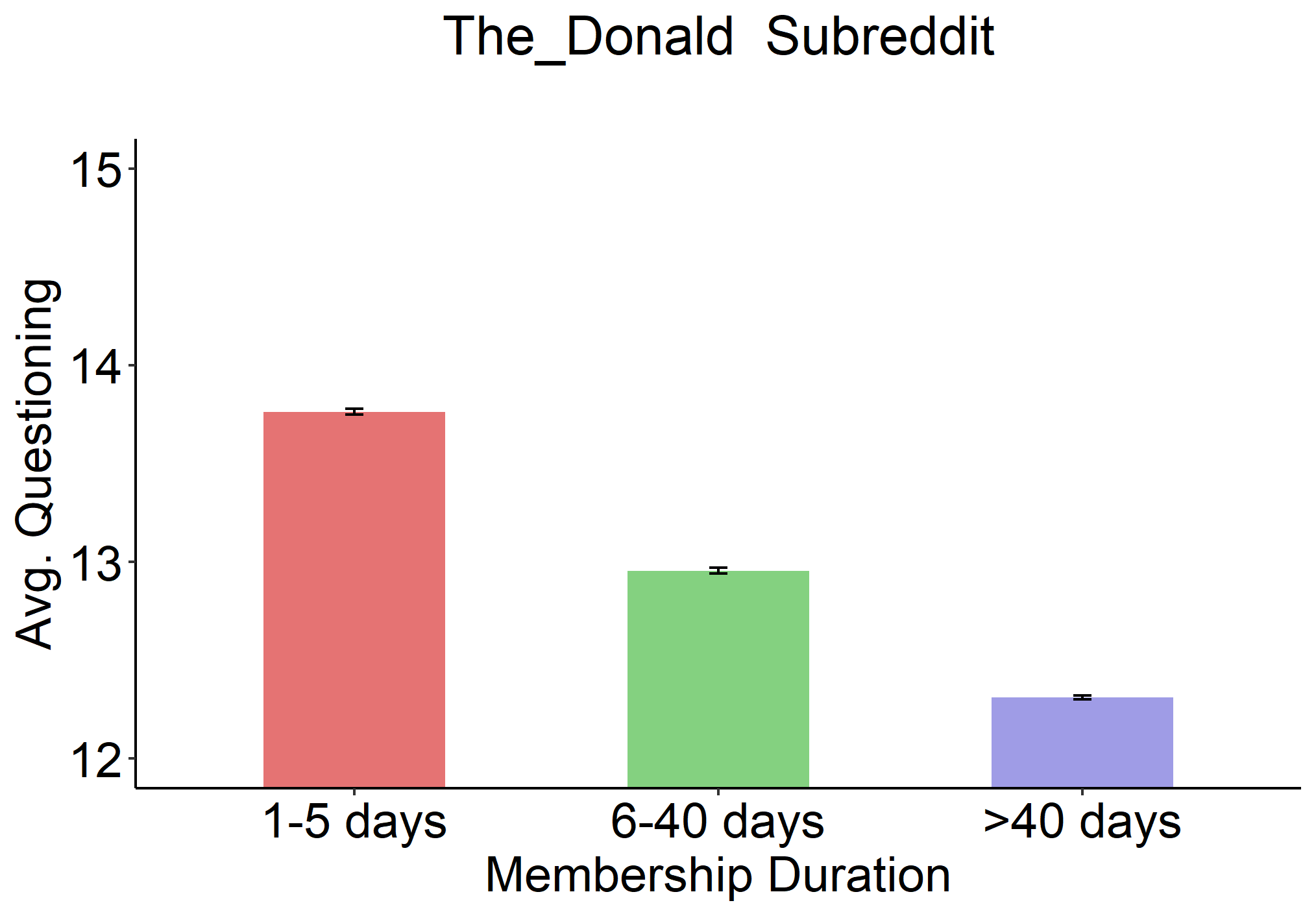 | 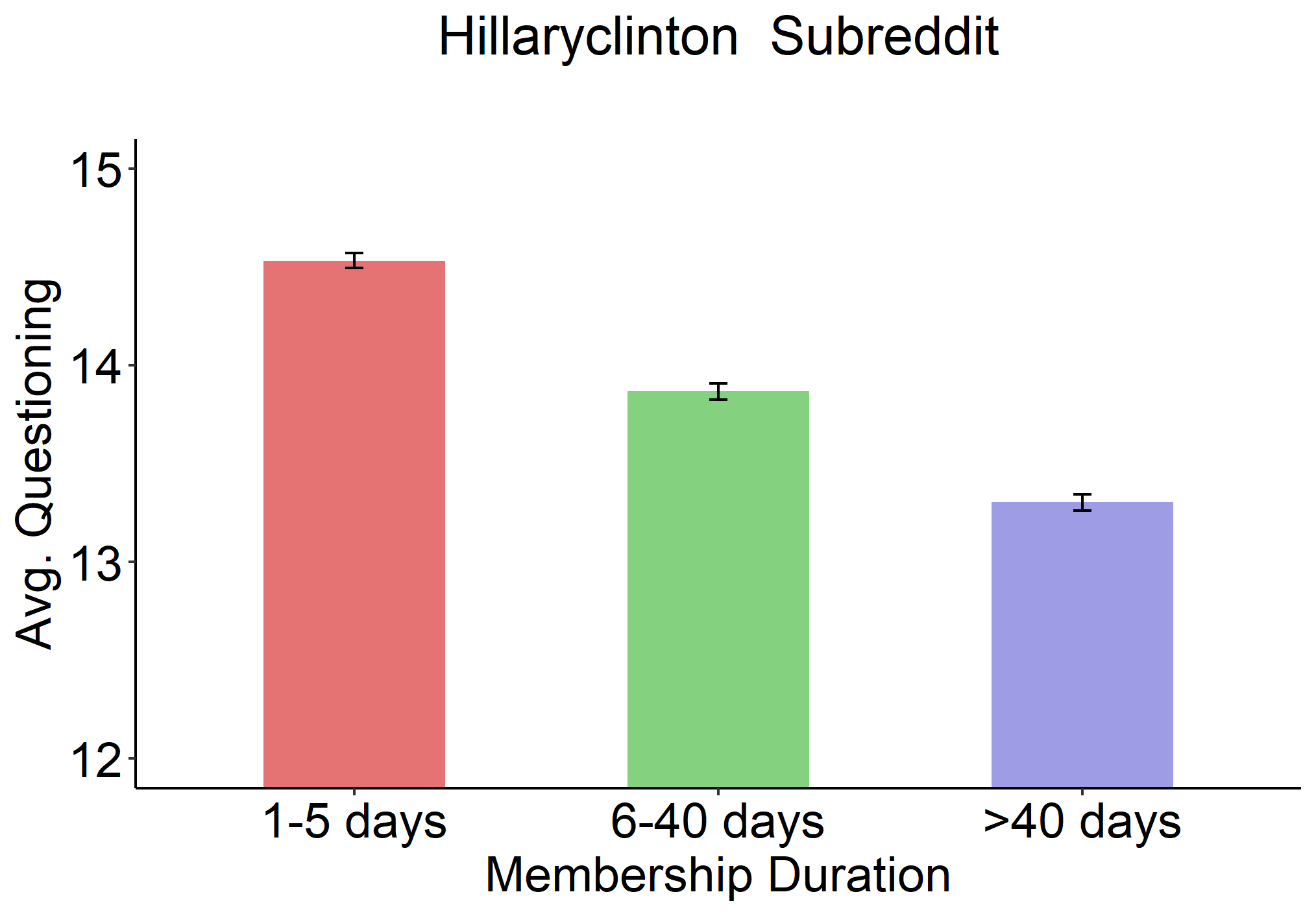 |
| --- | --- |

Figures S6a and S6b. Means of questioning for short-, medium- and long-term members of *The_Donald* and *hillaryclinton*.

## Study 3b Additional Methodological Notes and Robustness Check Analyses

**Samples.** Table S6 presents Study 3b sample sizes broken down by membership duration.

**Table S6.** Sample details broken down by duration of membership in the group (Study 3b)

|  | Stayed for 10-19 days | Stayed for 20-39 days | Stayed for >=40 days |
| --- | --- | --- | --- |
| Initial days in the group |  |  |  |
| *The_Donald* | 49,540 texts from 20,008 members | 69,192 texts from 14,492 members | 83,488 texts from 15,179 members |
| *hillaryclinton* | 5,685 texts from 1,780 members | 7,412 texts from 1,149 members | 9,608 texts from 1,360 members |
| Final days in the group |  |  |  |
| *The_Donald* | 46,678 texts from 19,572 members | 63,412 texts from 14,285 members | 230,464 texts from 15,480 members |
| *hillaryclinton* | 5,159 texts from 1,751 members | 6,746 texts from 1,144 members | 26,949 texts from 1,364 members |

**Analysis treating date as a fixed factor.** Unquestioning affiliation scores in Study 3 were computed using affiliation and cognitive processing scores that were standardized within the date on which each post was posted. This was done to account for the wide variation in when people posted on the group. For example, a user ⁠— say X ⁠— may have joined the group on election day (in November 2016). A different user ⁠— say Y⁠— may have joined the group 11 months before the election in January 2016. Analyzing raw LIWC scores would mean treating X’s first post (on election day) and Y’s first post (January 2016) in the same manner without accounting for the changing political context. Affiliation and cognitive processing LIWC scores were therefore standardized within each date in order to track individuals’ temporal changes while accounting for the context on any day. This approach made it easy to visualize the effects.

Another, arguably more straightforward, approach would be to treat date as a fixed effect instead of normalizing scores within each date. Doing so did not alter the findings. In the table below, we report estimates from mixed effects models examining changes in language after joining (left side of the table) and before leaving (right side of the table) the two groups. Accounting for the fixed effect of the date on which any comment was posted, we found a linear increase in unquestioning affiliation after joining (see positive coefficients corresponding to *nth day since joining*) and a similar decrease prior to leaving (see negative coefficients corresponding to *nth day before leaving*). The main article reports models based on standardizing the outcome because this approach makes it easier to visualize the effects.

Table S7. Estimates from mixed effects models predicting unquestioning affiliation expressed in language while controlling for the fixed effect of date

|  | Initial days after joining | | | | Final days prior to leaving | | | |
| --- | --- | --- | --- | --- | --- | --- | --- | --- |
|  | *The_Donald* | | *Hillaryclinton* | | *The_Donald* | | *Hillaryclinton* | |
| *Predictors* | *Estimates* | *p* | *Estimates* | *p* | *Estimates* | *p* | *Estimates* | *p* |
| (Intercept) | -5.076 | <0.001 | -6.200 | 0.010 | 0.189 | 0.621 | -7.272 | <0.001 |
| **Nth day since joining** | **0.009** | **<0.001** | **0.011** | **0.002** | **-** | **-** | **-** | **-** |
| **Nth day before leaving*** | **-** | **-** | **-** | **-** | **-0.004** | **<0.001** | **-0.003** | **0.001** |
| Comment date | 0.000 | <0.001 | 0.000 | 0.012 | -0.000 | 0.474 | 0.000 | <0.001 |
| **Random Effects** | | | | | | | | |
| σ^2^ | 2.09 | | 1.98 | | 2.16 | | 2.11 | |
| τ_00_ | 0.26 _author_id_ | | 0.22 _author_id_ | | 0.22 _author_id_ | | 0.21 _author_id_ | |
| ICC | 0.11 | | 0.10 | | 0.09 | | 0.09 | |
| N | 49679 _author_id_ | | 4289 _author_id_ | | 49337 _author_id_ | | 4259 _author_id_ | |
| Observations | 202220 | | 22705 | | 340554 | | 38854 | |
| **Marginal R^2^ / Conditional R^2^** | **0.001 / 0.113** | | **0.001 / 0.101** | | **0.001 / 0.093** | | **0.001 / 0.090** | |

*Note: * Nth day before joining* was negatively valenced. For example, an individual’s last active day in the group was coded as -1 and the day before that as -2. Also note that these estimates were obtained from a model in which date was entered as a fixed effect, which is different from the approach taken in the article.

**Analysis of members’ entire lifespan.** In the article and in the above analysis, we separately examined people’s initial and final days in a group. The graphs below (Fig. S7a-c) depict unquestioning affiliation scores over members’ normalized lifespan. Across the groups, the trajectory was generally shaped like an inverted-U, especially for long-term group members. For this group, expressions of unquestioning affiliation in language increased in members’ first days in the group and dropped prior to leaving. The patterns were weaker for the short-term members.

| 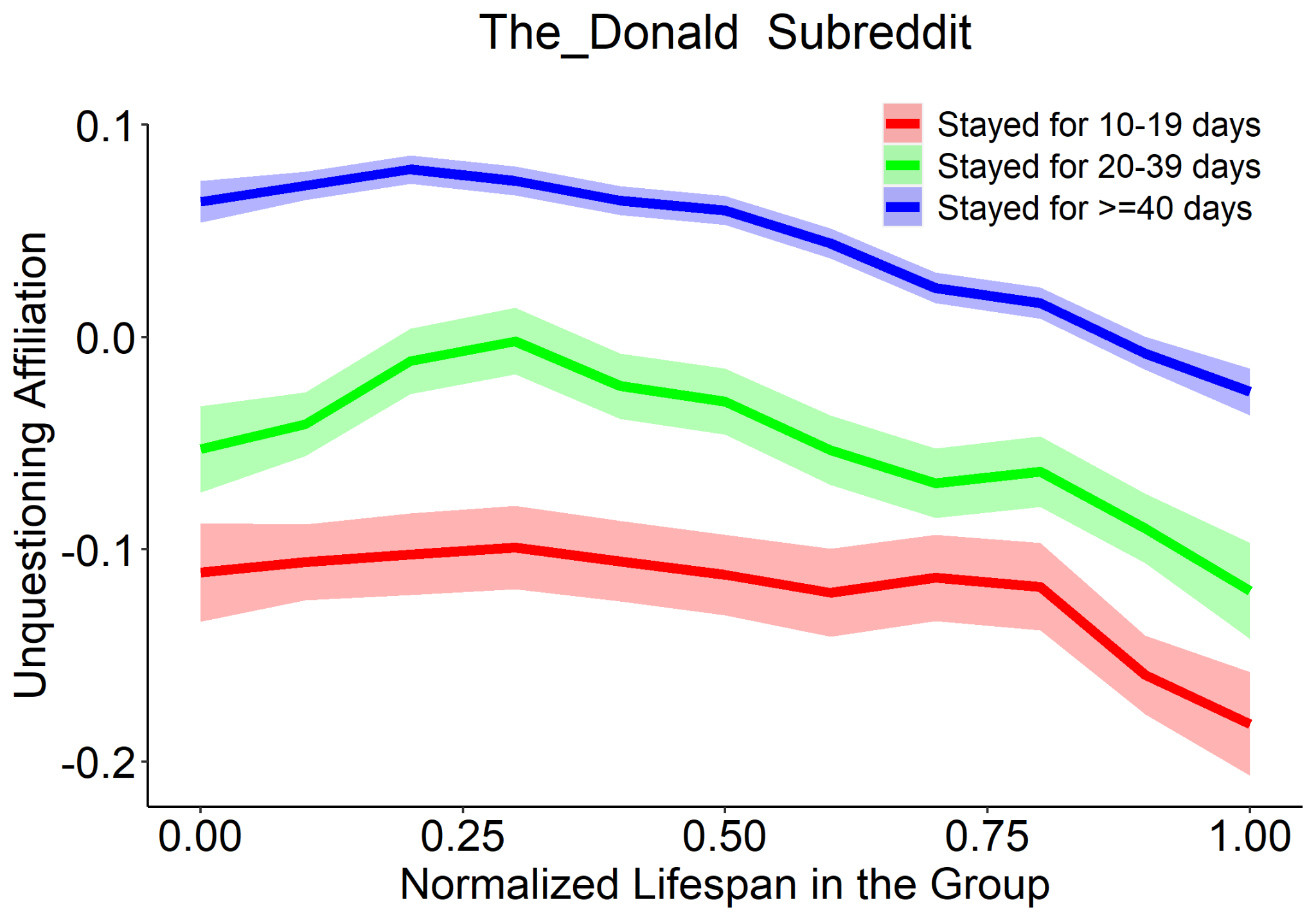 | 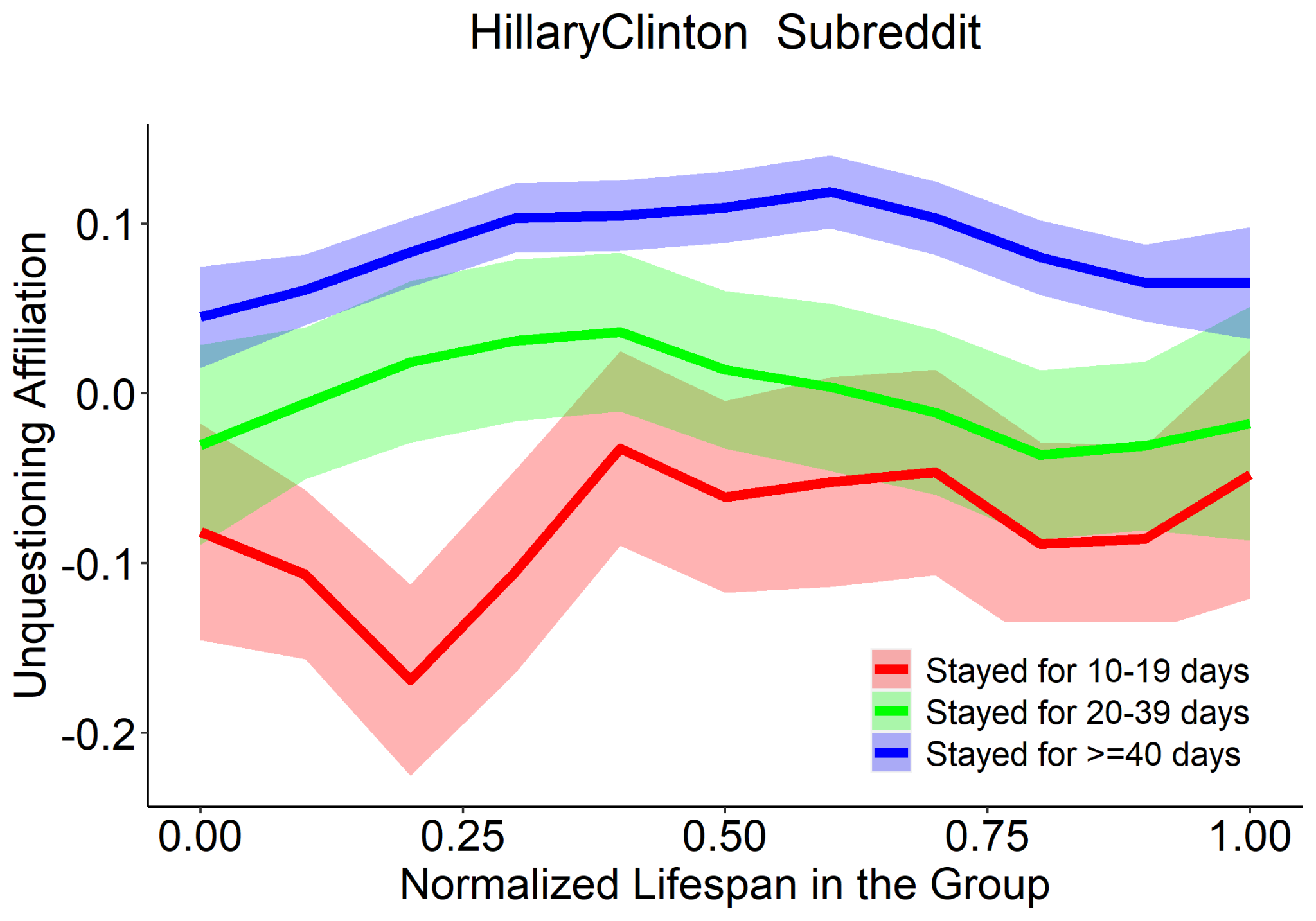 |
| --- | --- |
| 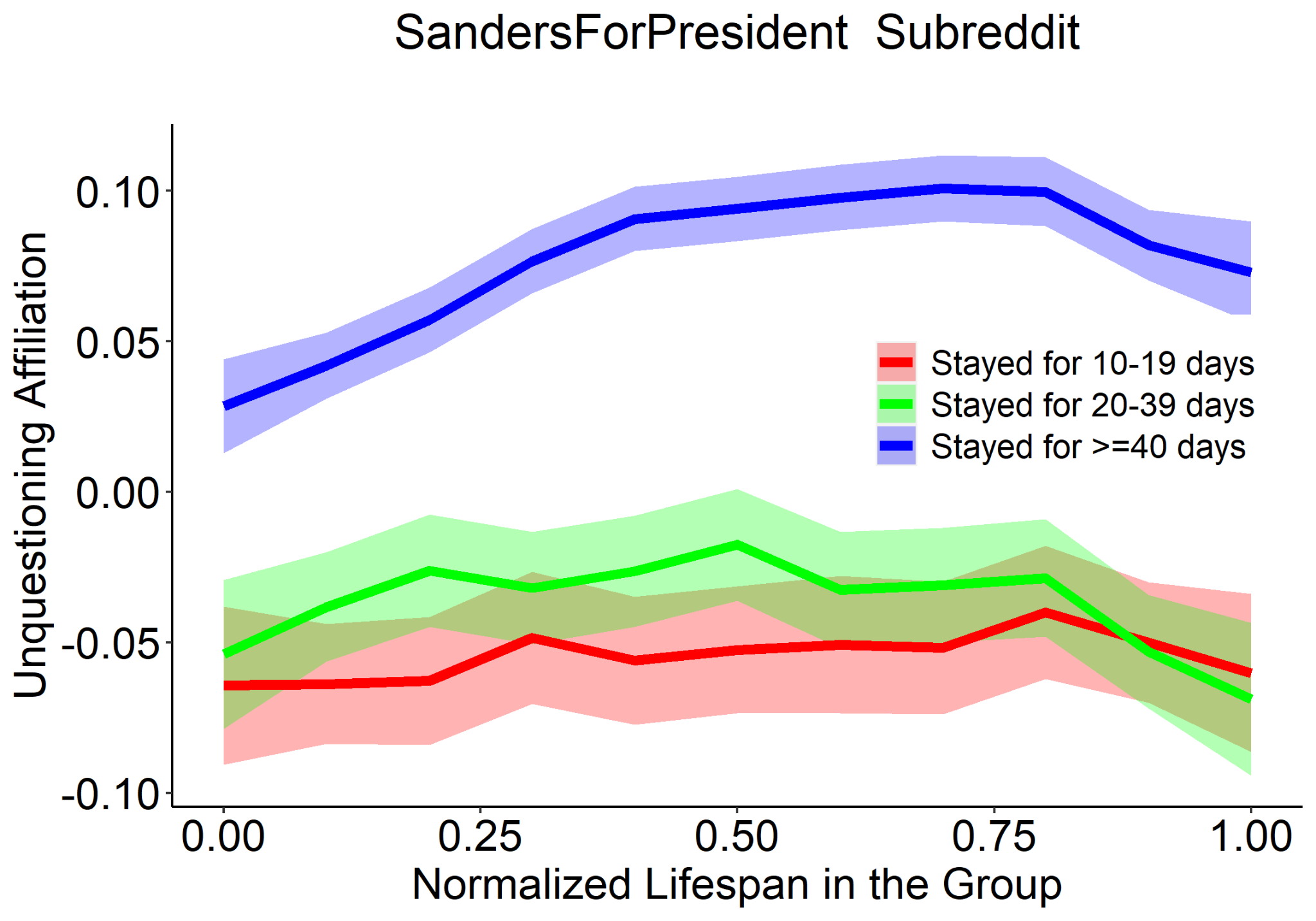 |  |

Figure S7a-S7c. Average unquestioning affiliation expressed in language over users’ normalized lifespan in the *The_Donald* (S7a), *hillaryclinton* (S7b), and *SandersForPresident* (S7c). The y-axis shows three-day rolling means.

**Separate analyses of affiliation and questioning.** We also separately assessed the temporal patterns reported in the main article for the two focal language indices. The general patterns in Figures S8a and S8b suggest that the use affiliation-related language tended to increase the longer people stayed in a group (*The_Donald*: *b* = .004, *t*(200754.8) = 5.19**;** *hillaryclinton*: *b* = .008, *t*(22516.4) = 3.31) and dropped prior to leaving the group (*The_Donald*: *b* = -.001, *t*(250865.4) = -6.12**;** *hillaryclinton* : *b* = -.001, *t*(29245.8) = -1.85, *p* = .06). Consistent with this, people’s language indicated a decrease in questioning after they joined the group (*The_Donald*: *b* = -.005, *t*(198510.7) = -6.63**;** *hillaryclinton*: *b* = -.003, *t*(22288.6) = -1.68, *p* = .09) and an increase in questioning as they approached their time of leaving (*The_Donald*: *b* = .003, *t*(278982.3) = 15.4**;** *hillaryclinton* : *b* = .003, *t*(32259.5) = 5.54; see Figures 9a and 9b). Two of the four predicted effects in *hillaryclinton* were only marginally significant. A possible explanation for the weak temporal effects in *hillaryclinton might* have to do with the effects of the 2016 elections. A large proportion of *hillaryclinton* members left the group immediately after the 2016 elections, and so shifts relating to individuals exiting the group (decrease in affiliation, increase in questioning) may be confounded with election effects (increase in affiliation, decrease in questioning).

| **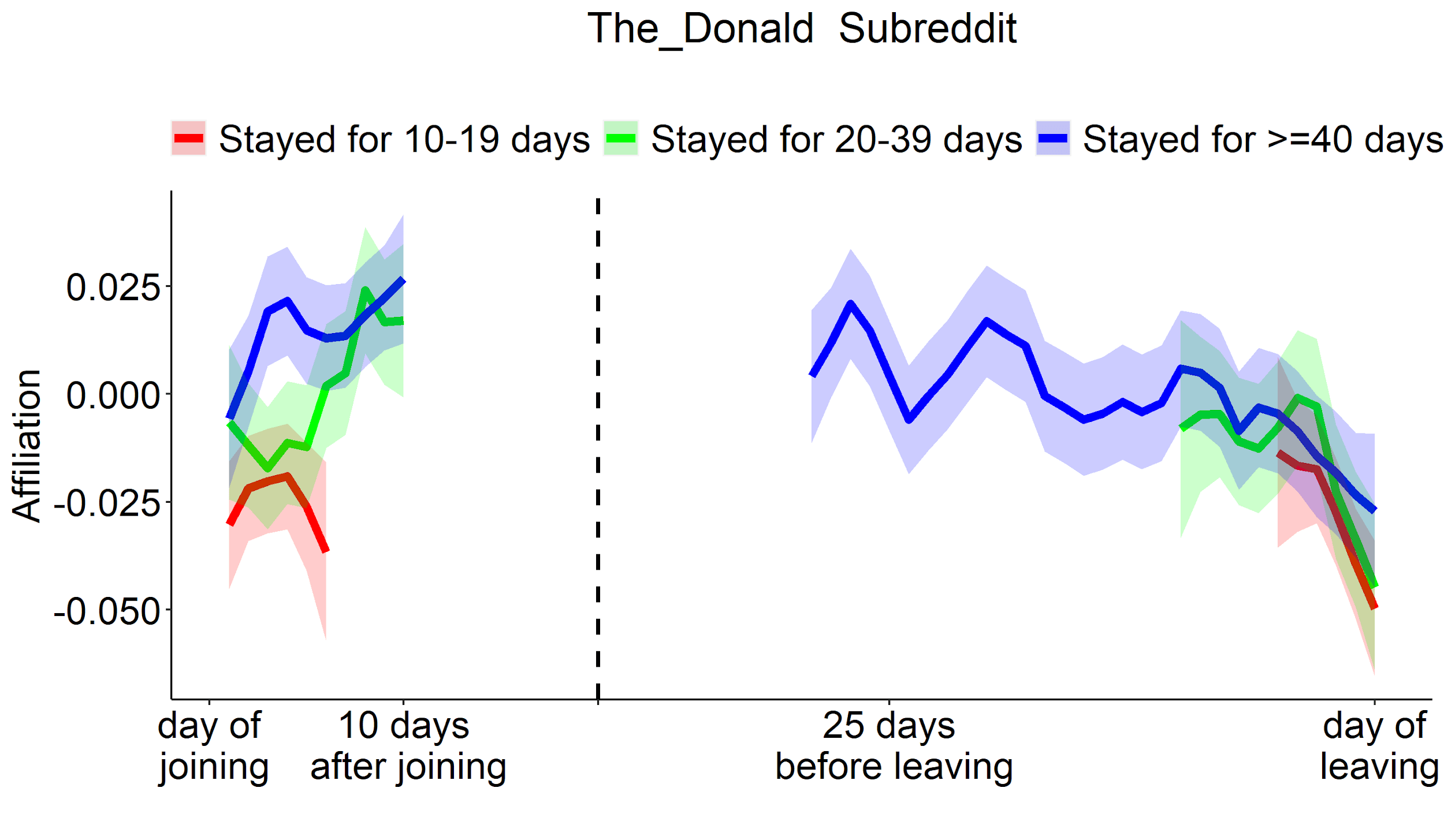** | 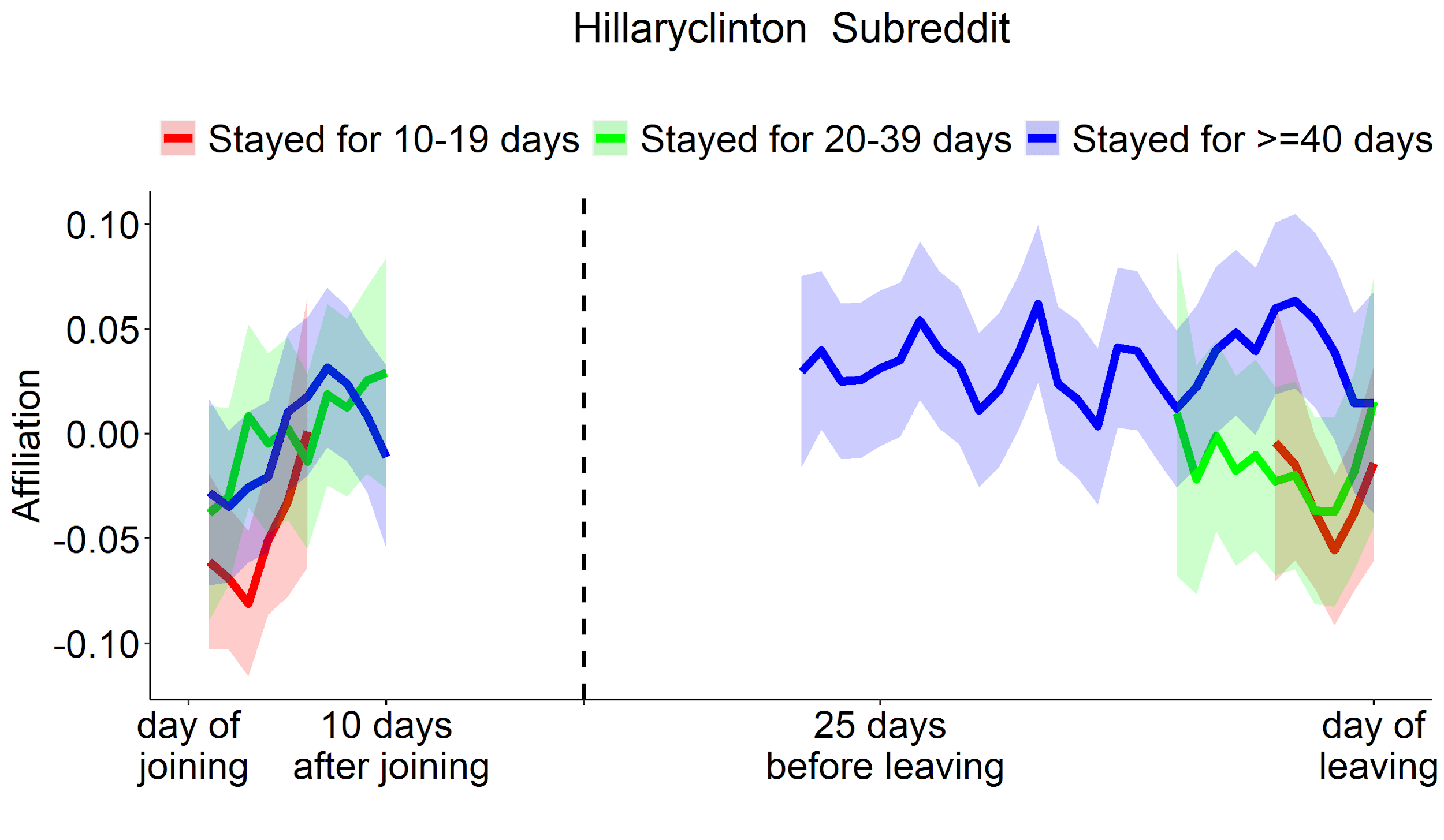 |
| --- | --- |

Figures S8a and S8b. Temporal effects on words relating to affiliation after joining and before leaving *The_Donald* (S8a) and *hillaryclinton* (S8b). The y-axis shows three-day rolling means of the affiliation scores, which were determined using LIWC’s affiliation dictionary.

| 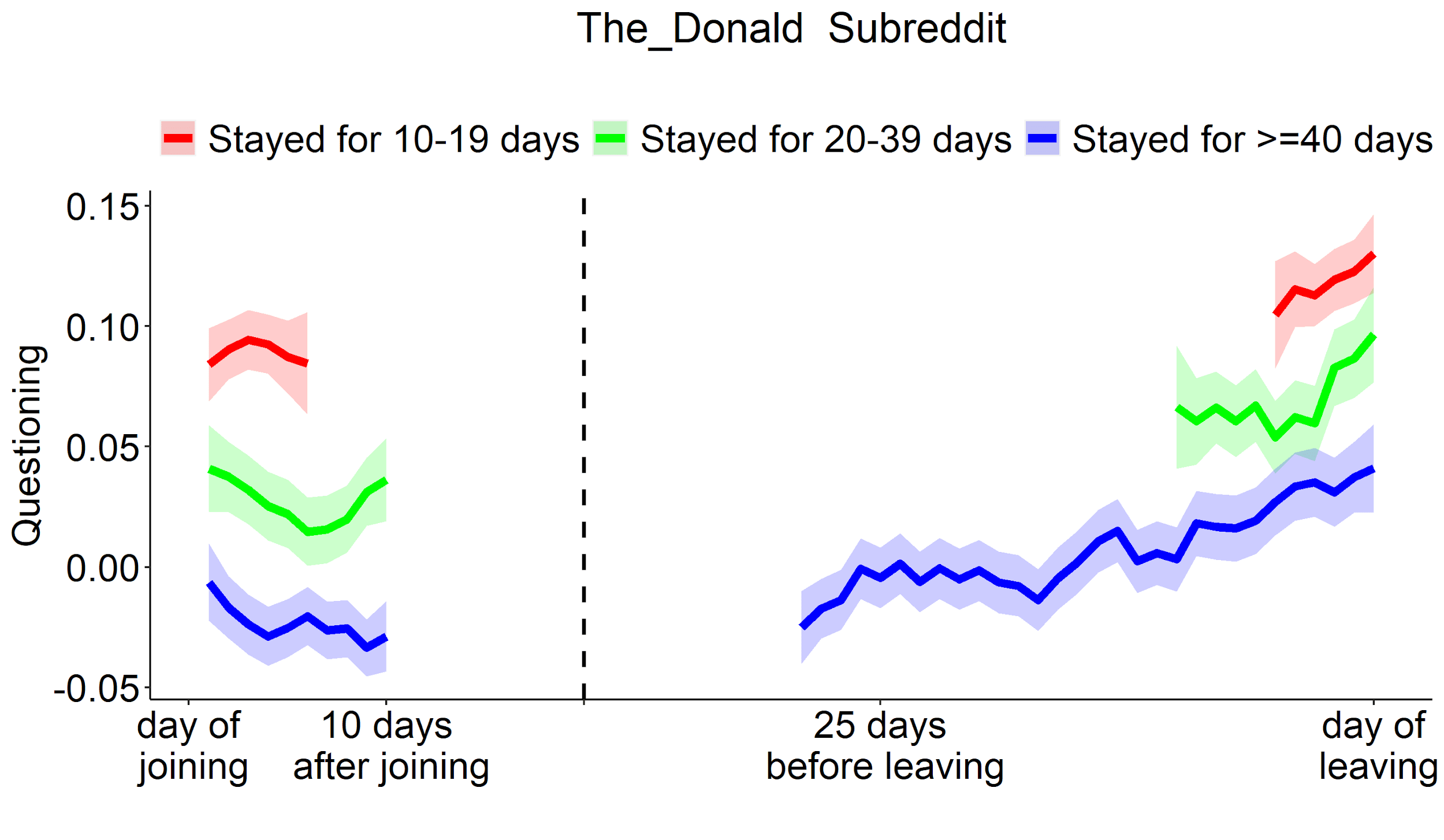 | 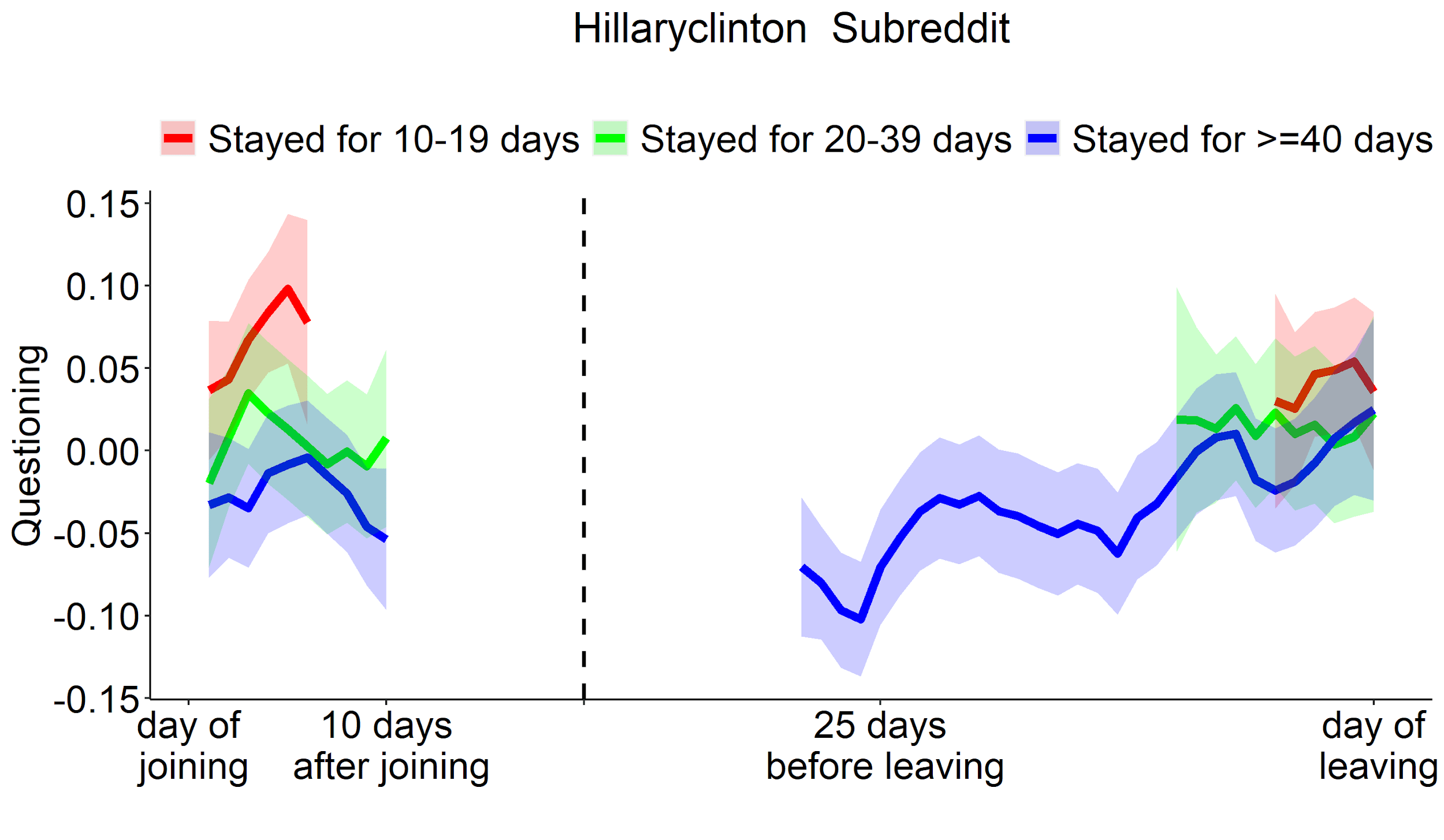 |
| --- | --- |

Figures S9a and S9b. Temporal effects on relating to questioning after joining and before leaving *The_Donald* (S9a) and *hillaryclinton* (S9b). The y-axis shows three-day rolling means of the questioning scores, which were determined using LIWC’s cognitive processing dictionary.

## Study 3 Replication in a sample of Bernie Sanders Supporters

**Study 3a.** Comments were extracted from a subreddit called *SandersForPresident.* The dataset included 795,004 comments posted between 5 December, 2013, to 31 December, 2018, by 142,686 users. The same exclusion criteria as in the Donald Trump and Hillary Clinton groups were applied. The method described in the article was used for measuring how much participants expressed unquestioning affiliation in their language.

Long-term group members used language conveying higher levels of unquestioning affiliation than short-term group members of the group: *F*(2, 142597) = 68.5; *d*_>40 vs. 1-5 days_ = .12 (see Fig. S10). Examining only comments from participants’ five days on the group produced similar effects. Long-term members used language reflecting higher levels of unquestioning affiliation even on their day of joining the group, *F*(2, 102899) = 7.24, *d*_>40 vs. 1-5 days_ = .06.


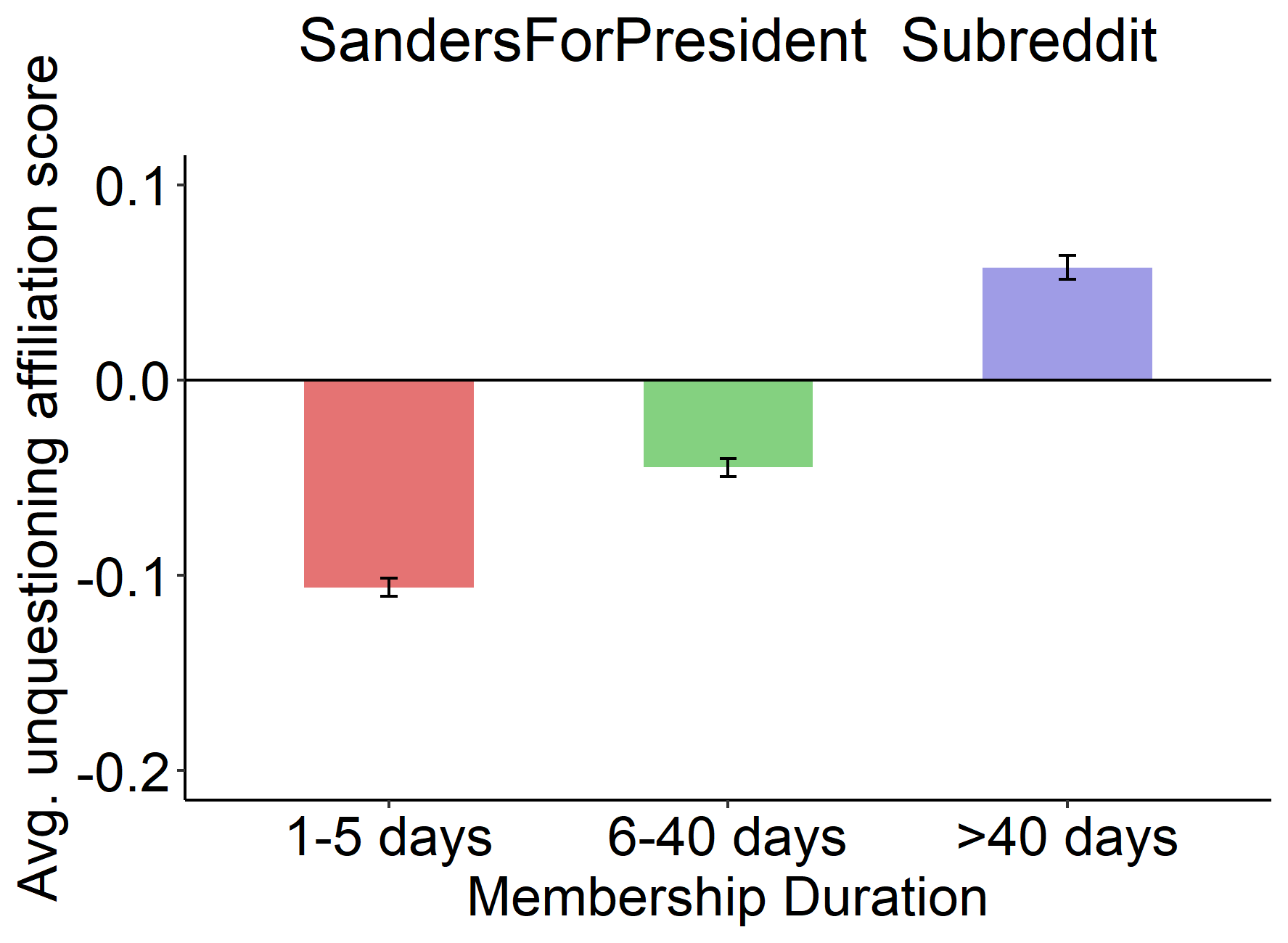


Figure S10. Average unquestioning affiliation scores for short-, medium- and long-term members of *SandersForPresident*. Group members who stayed in the group longer expressed more unquestioning affiliation in their language on average.

**Study 3b.** The analyses included texts from the initial days of 26,163 members and the final days of 26,110 members. Two mixed effects models accounting for random intercepts due to each person were tested. The first model sought to determine whether members expressed increasing levels of unquestioning affiliation in their language the longer they stayed in the group. A significant positive relationship between the number of days since joining and unquestioning affiliation emerged (*b* = .005, *t*(116821.5) = 3.20, *p* = .001**;** see Fig. S11). Another HLM model predicted unquestioning affiliation in members’ last days in the group. There was a significant drop in unquestioning affiliation expressed in language as people approached the day of exit from the group (*b* = -.003, *t*(139151.4) = -6.44).


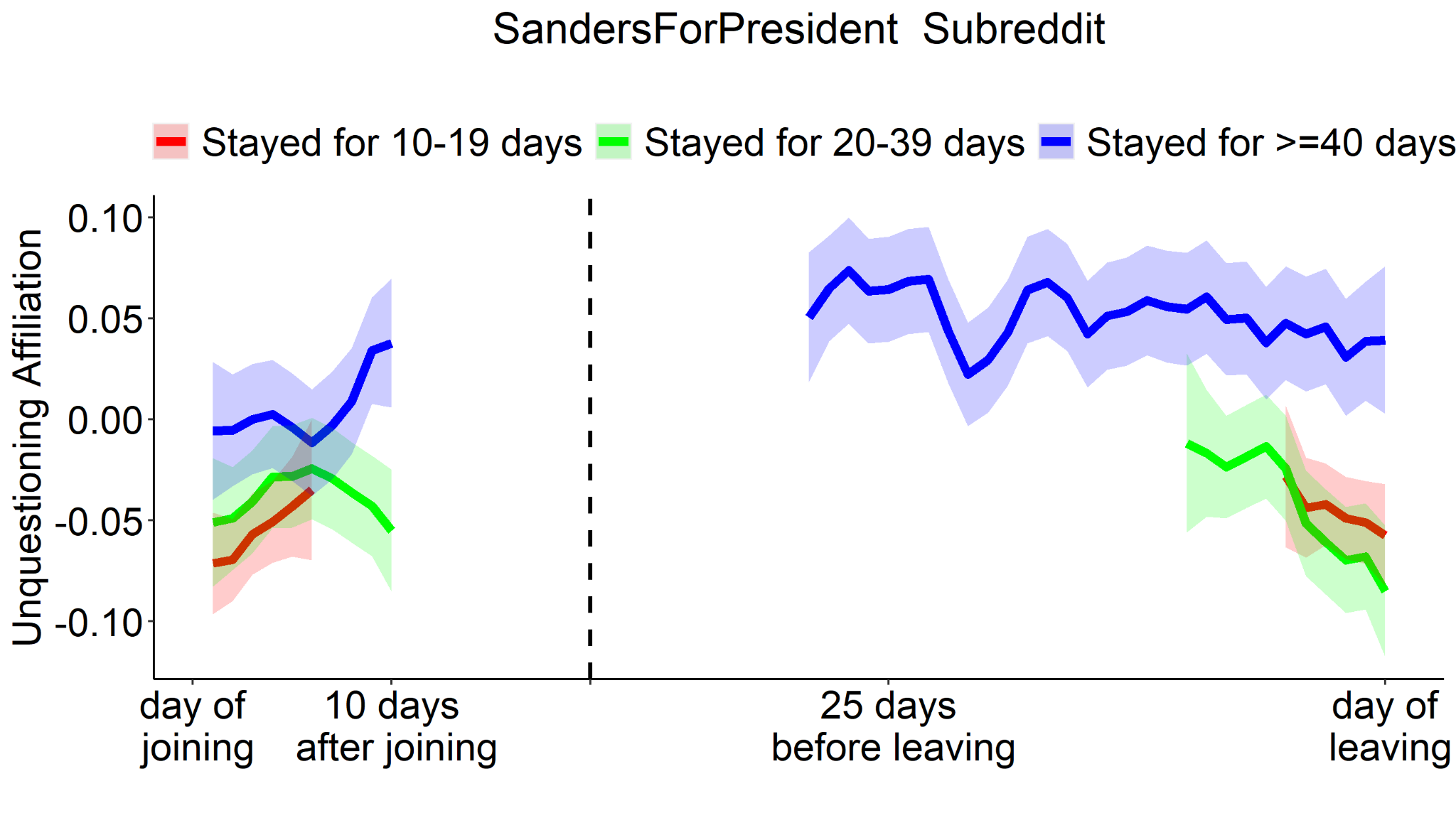


Figure S11. Temporal effects on levels of unquestioning affiliation expressed in language after joining and before leaving *SandersForPresident*. The y-axis shows rolling means of the unquestioning affiliation index over three days.

## We vs. Other Affiliation Words

The LIWC affiliation category includes we-words (first-person plural pronouns) such as *we*, *our*, and *us*, and also non-pronouns such as *together*, *pal*, and *buddy*. We-words used in conversations within groups indicate a collective frame of reference, showing that the speaker sees themselves as part of the group. People may not use we-words much when they are talking about a group in a lab setting or with an outsider. In such contexts, people may instead make direct references to the other group members and their relationships using words such as *buddy*, *love*, or *together*. It was expected that we-words would be better markers of identity in conversations within a group but that non-pronoun affiliation words would be more useful in other contexts. Below we present correlations and graphs comparing we-words and other affiliation words in the language analyzed in the three studies.

The correlations in Table S8 reveal that non-pronoun affiliation words outperformed we-words in Studies 1 and 2 wherein participants wrote about their group in relatively controlled settings.

**Table S8.** Correlations of self-reported indices with we-words vs. non-pronoun affiliation words in Studies 1-2.

|  | Language indices | Self-reported identity fusion with group | Self-reported pro-group behavior |
| --- | --- | --- | --- |
| Study 1a:  U.S.A.  (N = 247) | We-words | .1 | .11 |
|  | Non-pronoun affiliation words | .21*** | .24*** |
| Study 1b:  Religion  (N = 372) | We-words | .11* | .05 |
|  | Non-pronoun affiliation words | .10^†^ | .17*** |
| Study 1c:  College  (N = 250) | We-words | .03 | .07 |
|  | Non-pronoun affiliation words | .26*** | .33*** |
| Study 2  (N = 2161) | We-words | .05* | NA |
|  | Non-pronoun affiliation words | .08*** |  |

*Note.* * indicates *p* < .05. ** indicates *p* < .01. *** indicates *p* < .001. ^†^ indicates *p* <= .1

The graphs below show how much we-words vs. non-pronoun affiliation words from Reddit conversations in *The_Donald* predicted various outcomes. We-words outperformed non-pronoun affiliation words in predicting how long members stayed active in the group (Fig. S12a and S12b), joining and leaving the group (Fig. S13a and S13b).

| 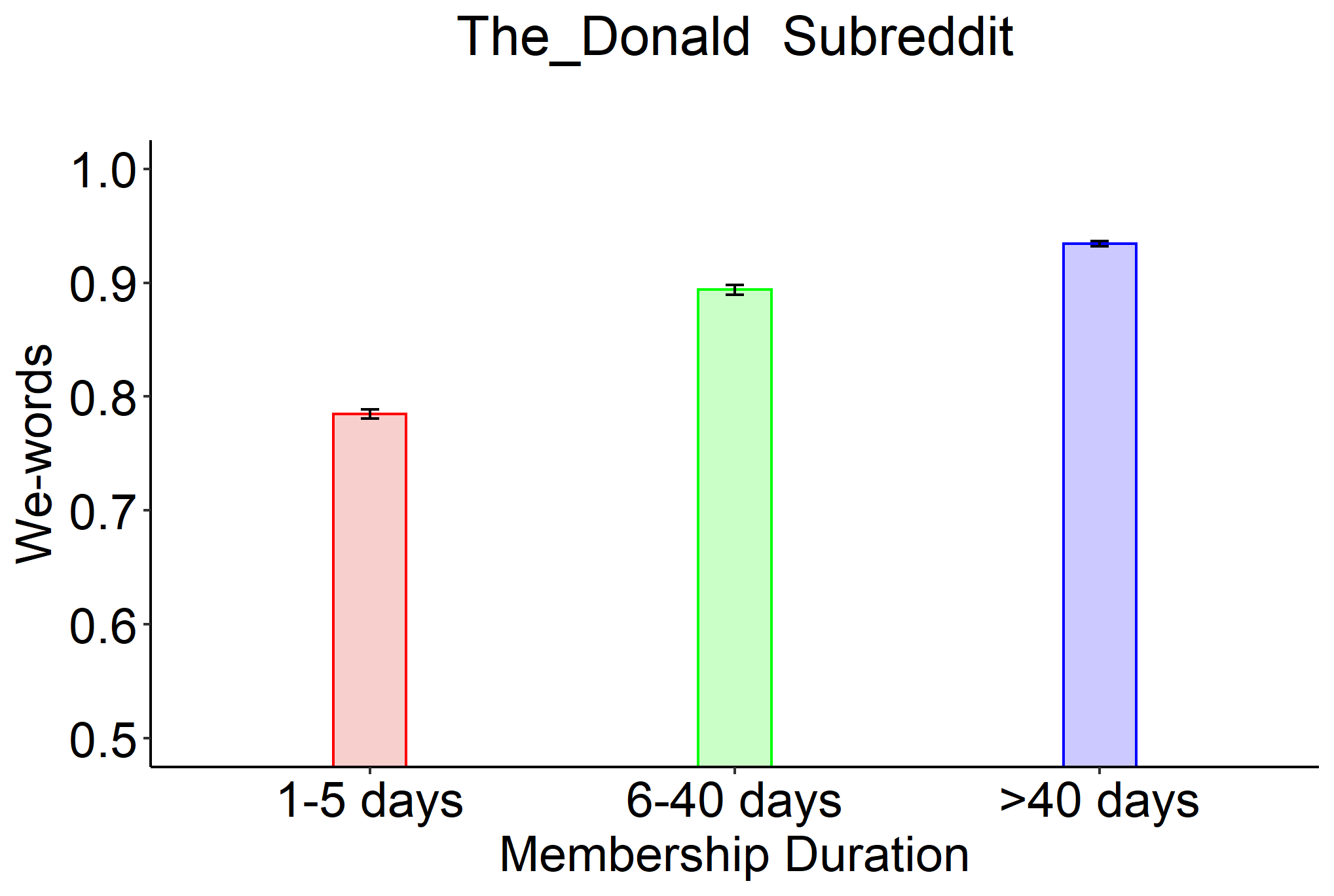 | 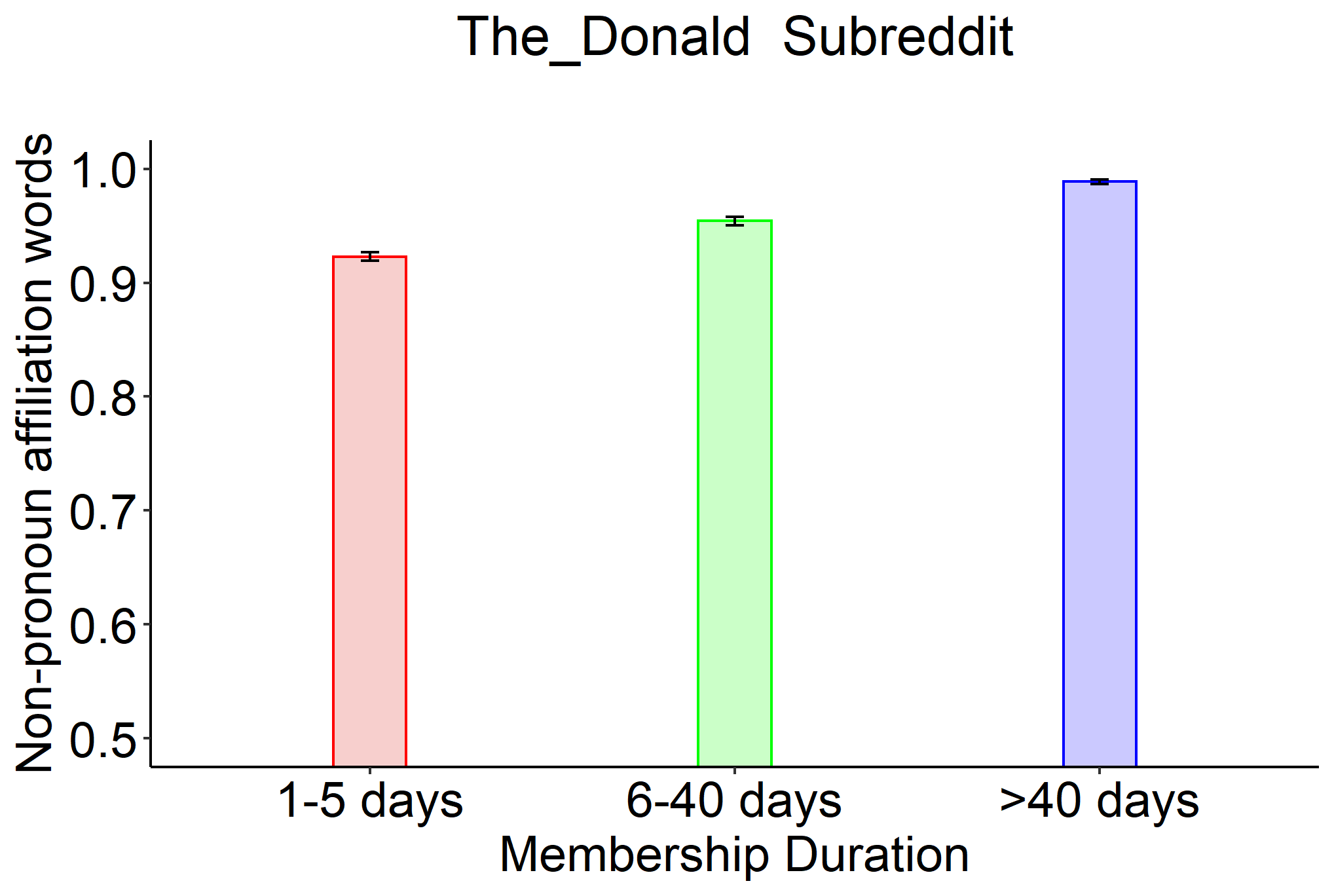 |
| --- | --- |

Figures S12a and S12b. Means of we-words (S12a) and non-pronoun affiliation words (S12b) for short-, medium- and long-term members of *The_Donald*.

| 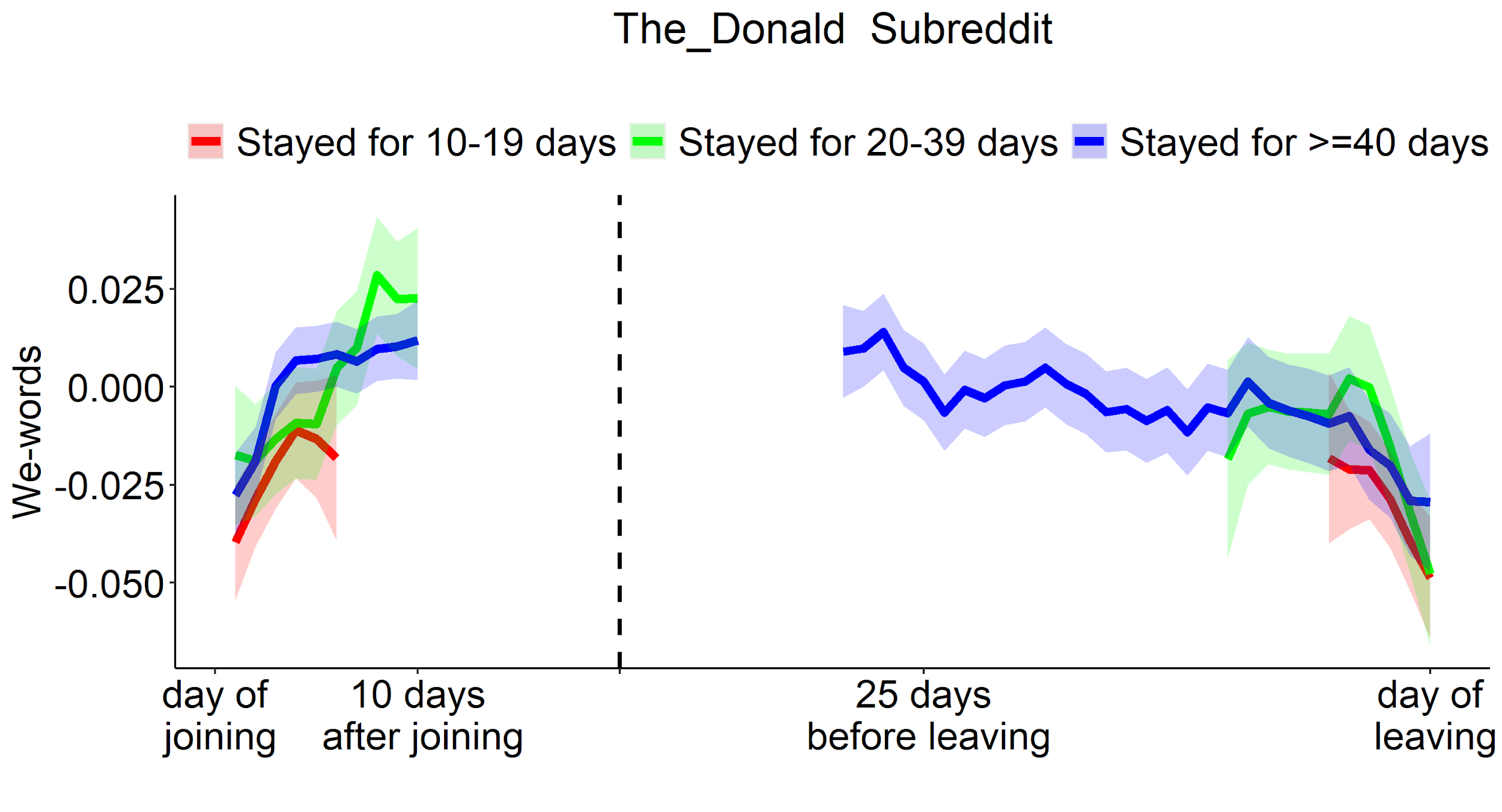 | 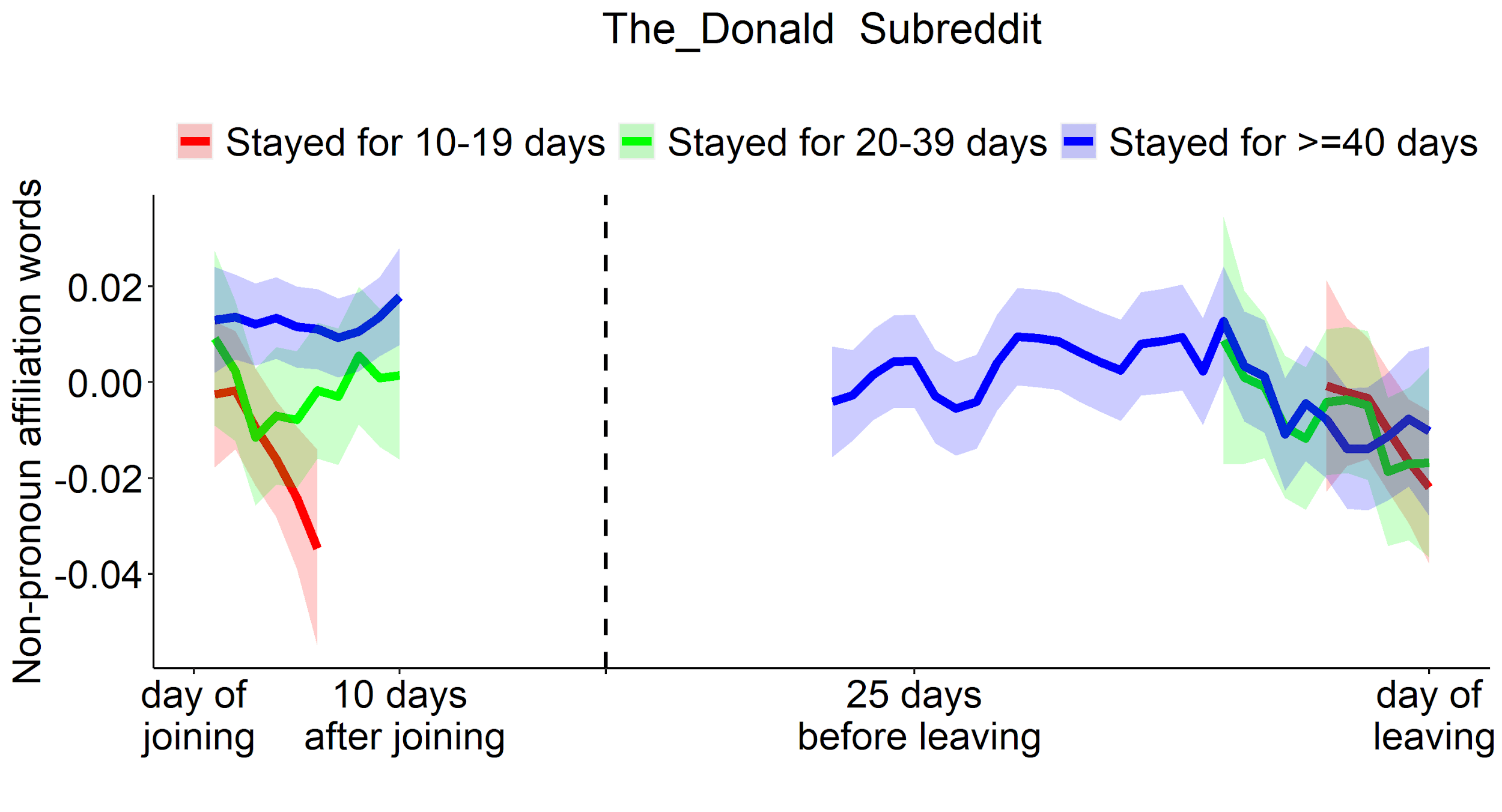 |
| --- | --- |

Figures S13a and S13b. Temporal effects on we-words (S13a) and non-pronoun affiliation words (S13b) in *The_Donald*.

## [Study 3](#_26in1rg) Analysis of Topics

The findings of the Reddit analysis suggest that people with strong identities used language indicating a greater sense of unquestioning connection. Exploring the topics that long-term vs short-term group members discussed could shed more light on the linguistic expressions of identity. This analysis was done using two approaches, both excluding stop words such as grammar words and other function words. First, for each user, the frequency of each word they used in their comments on their day of joining were computed. These were then correlated with the members’ active days in the group to determine the words most predictive of long-term membership. In the table below, the top row presents word clouds of the words most positively associated with staying in the group for a long time. The words in the second row were negatively correlated with long-term membership. As seen from the word clouds, people who went on to stay in the group longer talked about the candidates of their own party and the out-party. They also referred to elections (e.g., election, campaign, support, watch, poll, win) and positive words (e.g., love, great, hope). One of the words most predictive of long-term membership in The_Donald was “cuck”, which is a derogatory term used by Trump supporters and alt-right groups to insult mainstream conservatives who hold only moderately conservative positions, suggesting that group-specific jargon may provide a way of tracking group identity. The least used words in The_Donald were cognitive words (e.g., question, agree, understand) indicating that the user was questioning or working through some issues, and the only word negatively correlated with staying active in the hillaryclinton community was “email”, which likely refers to the email scandal that occurred in the days leading up to the 2016 election.

**Table S9.** Word clouds depticing words that were most positively (top row) and negatively (bottom row) correlated with long-term group membership

|  | The_Donald | hillaryclinton |
| --- | --- | --- |
| Words used **more** by long-term members | 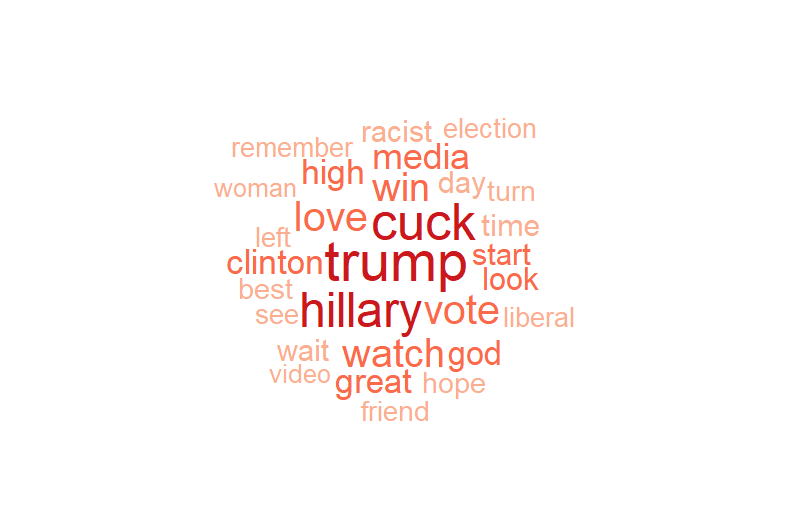 | 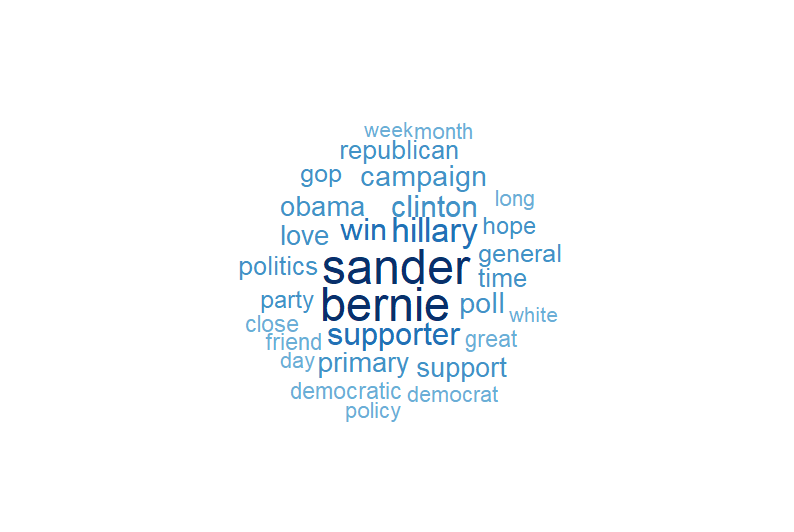 |
| Words used **less** by long-term members | 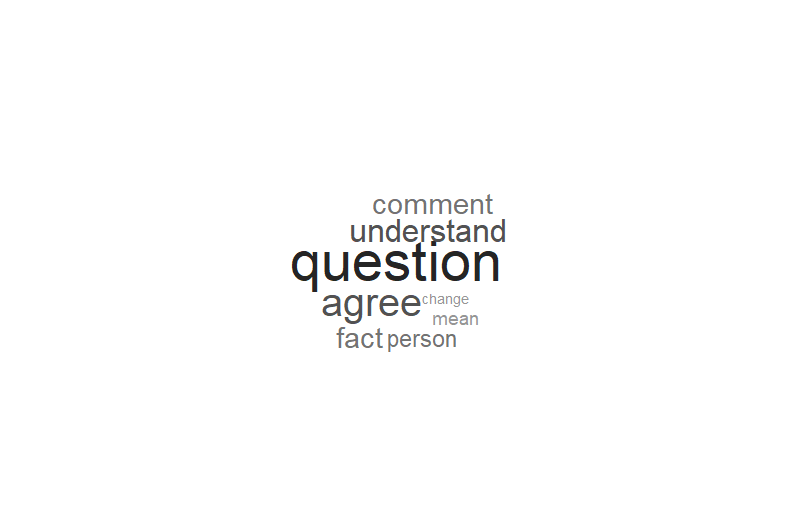 | 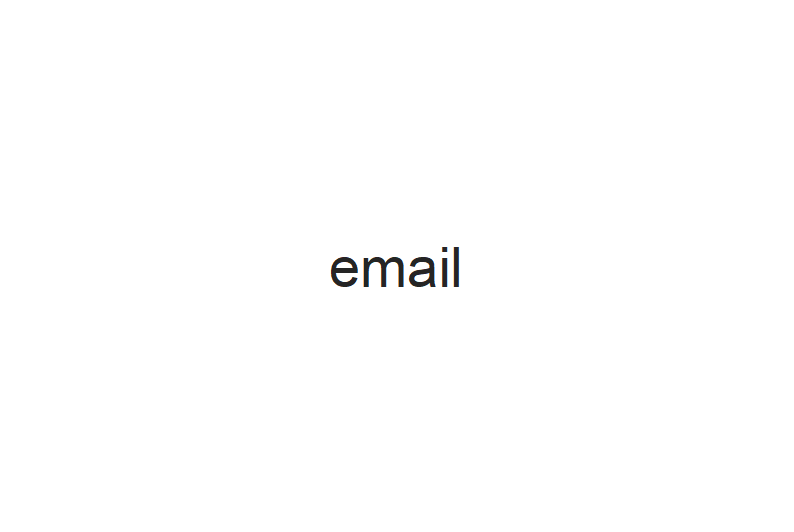 |

*Note*. The word “cuck” is a derogatory term short for weakness (as in cuckold) used by MAGA proponents to refer to “cuckservatives” who are often middle-of-the-road conservatives.

To further explore the topics used by long- versus short- term members, we conducted a topic analysis using the Meaning Extraction Method (Chung & Pennebaker, 2008). Again, long-term members talked about elections, candidates, and campaigning. One of the top topics in the Trump community included a focus on their campaign slogan “Make America Great Again”. Long-term members also talked about the news and related media, indicating that they are perhaps more politically engaged. A recurring theme that was negatively correlated with long-term membership was related to working through or making sense of various issues (see right-most column of Table S10)..

**Table S10.** Topics that were most strongly correlated with membership duration in the Trump (top row) and Clinton (bottom row) communities

| Topics positively correlated with membership duration | | | | Topics negatively correlated with membership duration |
| --- | --- | --- | --- | --- |
| *(a) Candidates & elections*  *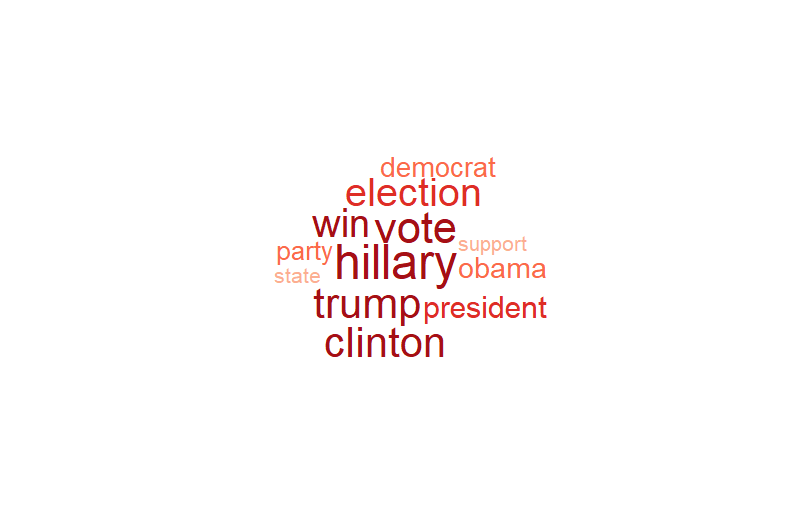* | *(b) MAGA**  *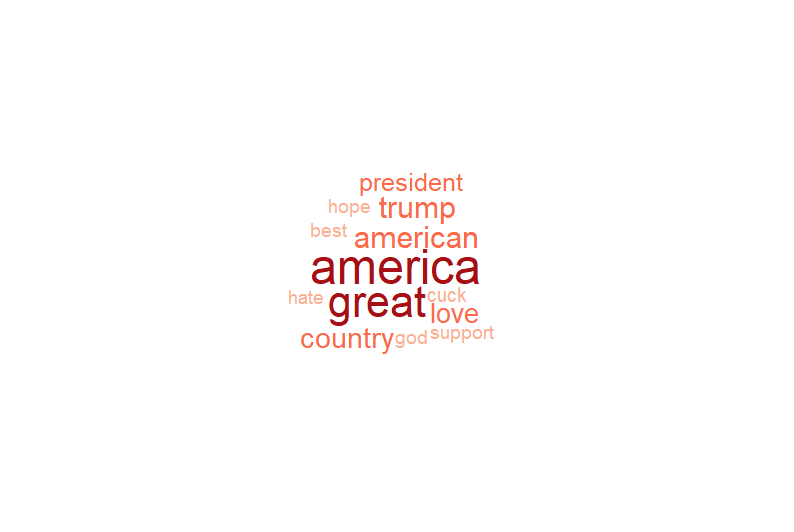* | *(c) Gender & family*  *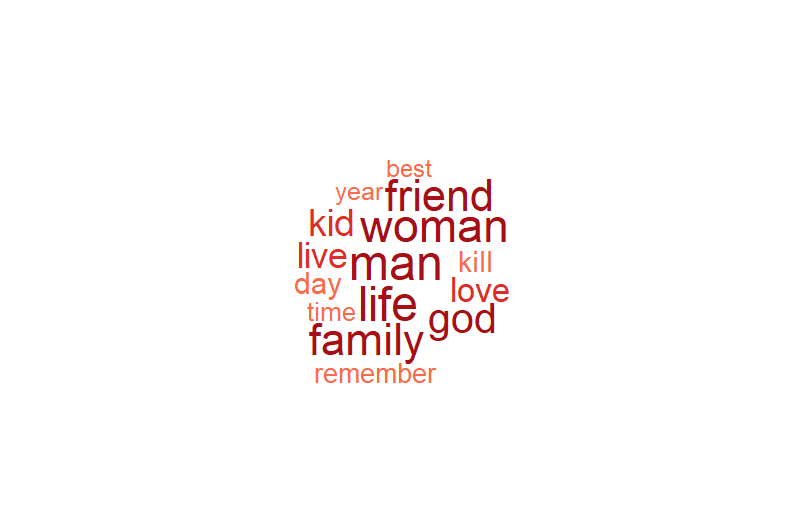* | *(d) News media*  *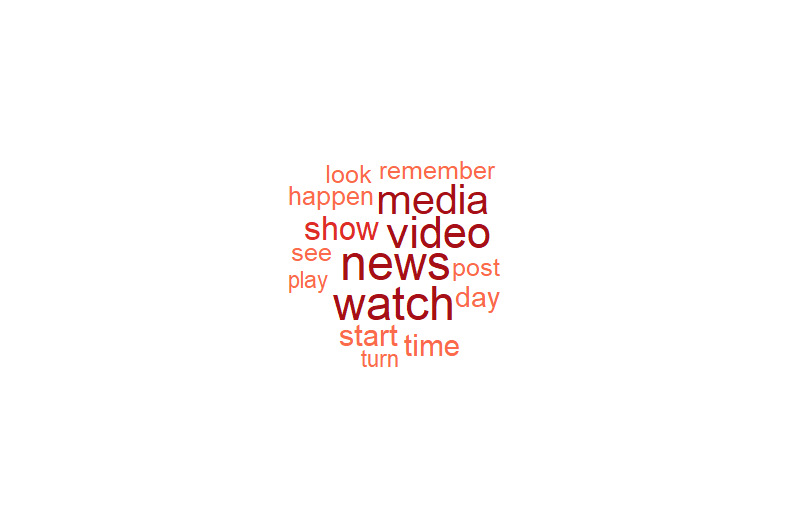* | *(e) Working through*  *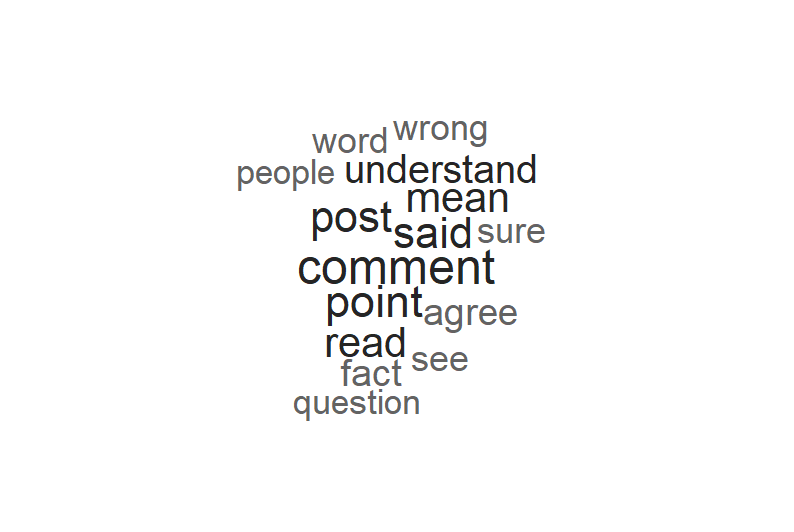* |
| *(a) Candidates & campaign*  *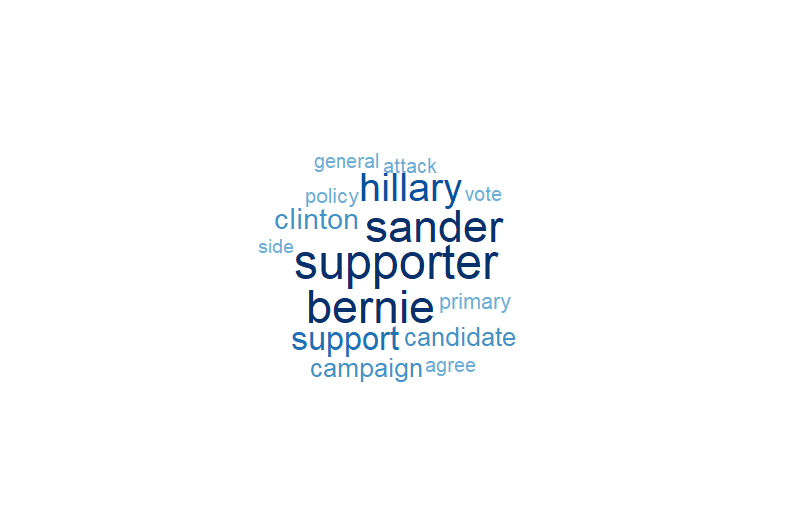* | *(b) News & time*  *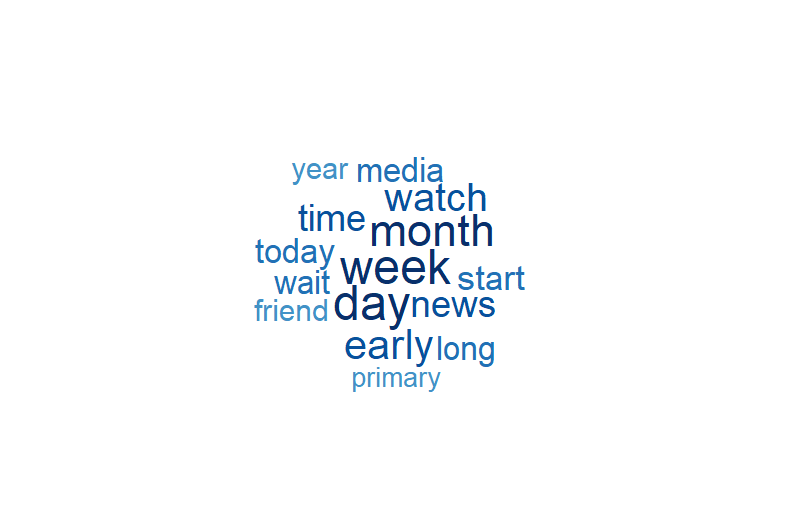* | *(c) Obama tenure*  *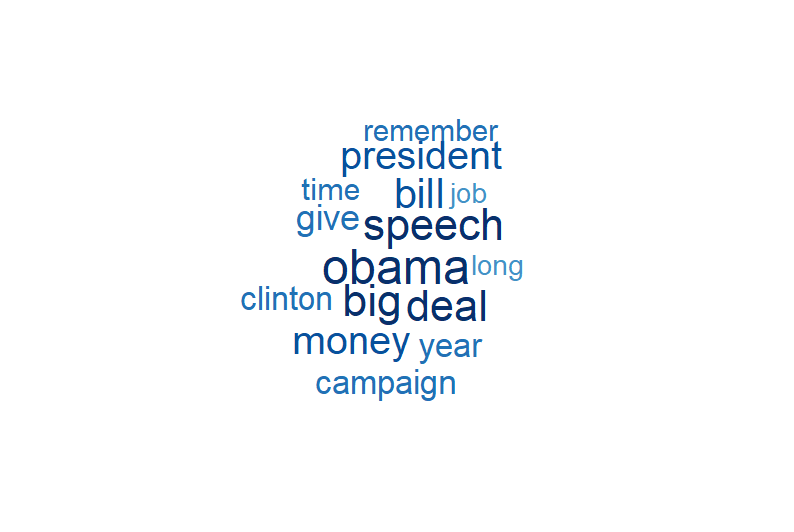* | *(d) Parties & elections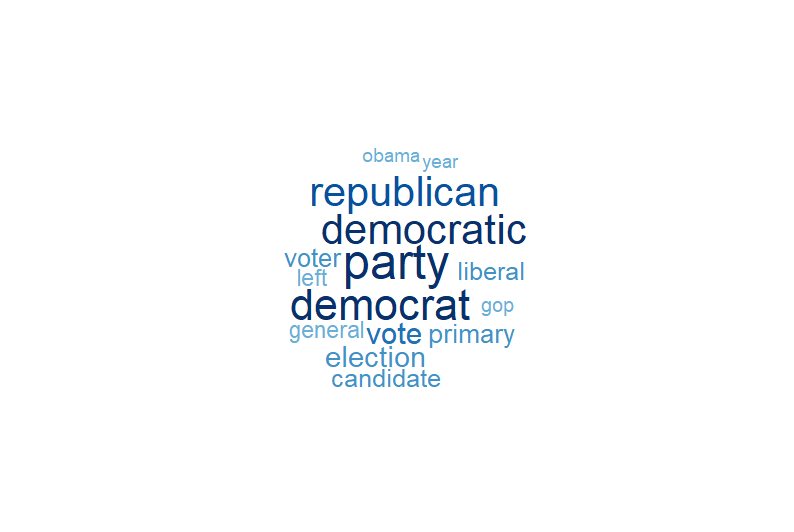* | *(e) Working through*  *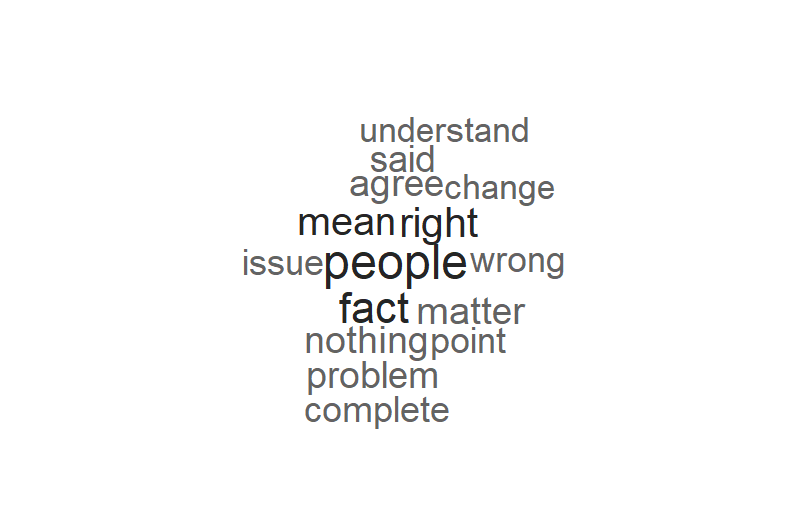* |

*Note. **Stop words or common function words (including *make* and *again*) were excluded in this analysis to focus on the content of text

## References

Boyd, R. L. (2017). Psychological text analysis in the digital humanities. In *Data analytics in digital humanities* (pp. 161-189). Springer, Cham.

Boyd, R. L., Blackburn, K. G., & Pennebaker, J. W. (2020). The narrative arc: Revealing core narrative structures through text analysis. *Science Advances*, *6*(32), eaba2196. https://doi.org/10.1126/sciadv.aba2196

Chung, C. K., & Pennebaker, J. W. (2008). Revealing dimensions of thinking in open-ended self-descriptions: An automated meaning extraction method for natural language. *Journal of research in personality*, *42*(1), 96-132.

[Cortina, J. M., & Landis, R. S. (2008). When small effect sizes tell a big story, and when large effect sizes don’t. *Statistical and Methodological Myths and Urban Legends: Doctrine, Verity and Fable in the Organizational and Social Sciences*, 287–308. https://doi.org/10.4324/9780203867266](https://www.zotero.org/google-docs/?broken=kXlBHm)

Goldenberg, A., & Gross, J. J. (2020). Digital emotion contagion. *Trends in Cognitive Sciences*, *24*(4), 316-328.

[Hamilton, W. L., Zhang, J., Danescu-Niculescu-Mizil, C., Jurafsky, D., & Leskovec, J. (2017). *Loyalty in Online Communities*. https://doi.org/10.1080/10826070701360368](https://www.zotero.org/google-docs/?broken=LAHj1f)

Haslam, C., Jetten, J., Cruwys, T., Dingle, G. A., & Haslam, S. A. (2018). *The new psychology of health: Unlocking the social cure*. Routledge.

Hogg, M. A. (2007). Uncertainty–Identity Theory. In *Advances in Experimental Social Psychology* (Vol. 39, pp. 69–126). Elsevier. https://doi.org/10.1016/S0065-2601(06)39002-8

Hornsey, M. J. (2008). Social Identity Theory and Self-categorization Theory: A Historical Review: Social Identity Theory and Self-categorization Theory. *Social and Personality Psychology Compass*, *2*(1), 204–222. <https://doi.org/10.1111/j.1751-9004.2007.00066.x>

[Kern, M. L., Park, G., Eichstaedt, J. C., Schwartz, H. A., Sap, M., Smith, L. K., & Ungar, L. H. (2016). Gaining insights from social media language. *Psychological Methods*, *21*(4), 507–525. https://doi.org/10.1037/met0000091 T4 - Methodologies and challenges PM - 27505683 M4 - Citavi](https://www.zotero.org/google-docs/?broken=cmGAL1)

Klein, O., Spears, R., & Reicher, S. (2007). Social identity performance: Extending the strategic side of SIDE. *Personality and Social Psychology Review*, *11*(1), 28-45.

[Matz, S. C., Gladstone, J. J., & Stillwell, D. (2017). In a World of Big Data, Small Effects Can Still Matter. *Psychological Science*. https://doi.org/10.1177/0956797617697445](https://www.zotero.org/google-docs/?broken=LHvrNe)

Seraj, S., Blackburn, K. G., & Pennebaker, J. W. (2021). Language left behind on social media exposes the emotional and cognitive costs of a romantic breakup. *Proceedings of the National Academy of Sciences*, *118*(7).

Smith, E. R., & Mackie, D. M. (2015). Dynamics of group-based emotions: Insights from intergroup emotions theory. *Emotion Review*, *7*(4), 349-354.

[Steers, R. M. (1977). Antecedents and Outcomes of Organizational Commitment. *Administrative Science Quarterly*, *22*(1), 46–56. JSTOR. https://doi.org/10.2307/2391745](https://www.zotero.org/google-docs/?broken=RlVF4H)

Strenger, J. E., Goldenberg, A., Saguy, T., & Halperin, E. (2020). Differentiation from the ideological out-group as reference for the in-group’s emotions in conflict.

[Swann, W. B., Buhrmester, M. D., Gómez, A., Jetten, J., Bastian, B., Vázquez, A., Ariyanto, A., Besta, T., Christ, O., Cui, L., Finchilescu, G., González, R., Goto, N., Hornsey, M., Sharma, S., Susianto, H., & Zhang, A. (2014). What makes a group worth dying for? Identity fusion fosters perception of familial ties, promoting self-sacrifice. *Journal of Personality and Social Psychology*, *106*(6), 912–926. https://doi.org/10.1037/a0036089](https://www.zotero.org/google-docs/?broken=DKZaP4)

[Tajfel, H., & Turner, J. (1979). An integrative theory of intergroup conflict. In *The Social Psychology of Intergroup Relations* (pp. 33–47).](https://www.zotero.org/google-docs/?broken=QGQWPy)

[Walton, G. M., & Cohen, G. L. (2007). A question of belonging: Race, social fit, and achievement. *Journal of Personality and Social Psychology*, *92*(1), 82–96. https://doi.org/10.1037/0022-3514.92.1.82](https://www.zotero.org/google-docs/?broken=isVtp8)

[Zimmer, M. (2018). Addressing Conceptual Gaps in Big Data Research Ethics: An Application of Contextual Integrity. *Social Media + Society*, *4*(2), 2056305118768300. https://doi.org/10.1177/2056305118768300](https://www.zotero.org/google-docs/?broken=xKN7Gq)
